# A novel triple combination of pharmacological chaperones improves F508del-CFTR correction

**DOI:** 10.1038/s41598-018-29276-y

**Published:** 2018-07-30

**Authors:** Graeme W. Carlile, Qi Yang, Elizabeth Matthes, Jie Liao, Stevo Radinovic, Carol Miyamoto, Renaud Robert, John W. Hanrahan, David Y. Thomas

**Affiliations:** 1 Cystic Fibrosis Translational Research Center, Department of Biochemistry McGill University Montreal Quebec Canada, H3G 1Y6 Montreal, Quebec Canada; 2 Cystic Fibrosis Translational Research Center, Department of Physiology McGill University Montreal Quebec Canada, H3G 1Y6 Montreal, Quebec Canada; 3 National Research Council, Biotechnology Research Institute, 6100 Royalmount Ave, H4P 2R2 Montreal, Quebec Canada

## Abstract

Pharmacological chaperones (e.g. VX-809, lumacaftor) that bind directly to F508del-CFTR and correct its mislocalization are promising therapeutics for Cystic Fibrosis (CF). However to date, individual correctors provide only ~4% improvement in lung function measured as FEV1, suggesting that multiple drugs will be needed to achieve substantial clinical benefit. Here we examine if multiple sites for pharmacological chaperones exist and can be targeted to enhance the rescue of F508del-CFTR with the premise that additive or synergistic rescue by multiple pharmacological chaperones compared to single correctors indicates that they have different sites of action. First, we found that a combination of the pharmacological chaperones VX-809 and RDR1 provide additive correction of F508del-CFTR. Then using cellular thermal stability assays (CETSA) we demonstrated the possibility of a third pharmacologically important site using the novel pharmacological chaperone tool compound 4-methyl-N-[3-(morpholin-4-yl) quinoxalin-2-yl] benzenesulfonamide (MCG1516A). All three pharmacological chaperones appear to interact with the first nucleotide-binding domain (NBD1). The triple combination of MCG1516A, RDR1, and VX-809 restored CFTR function to >20% that of non-CF cells in well differentiated HBE cells and to much higher levels in other cell types. Thus the results suggest the presence of at least three distinct sites for pharmacological chaperones on F508del-CFTR NBD1, encouraging the development of triple corrector combinations.

## Introduction

The cystic fibrosis (CF) transmembrane conductance regulator (CFTR) is an anion channel expressed at the apical surface of secretory epithelia in the pancreas, intestine, exocrine glands and in lung^1,2^. Loss-of-function mutations in the *cftr* gene cause the lethal autosomal recessive disease cystic fibrosis (CF)^3^. Many of the >2000 known mutations in CFTR cause disease (https://cftr2.org) with the most common mutation a loss of a phenylalanne at position 508, in the first nucleotide binding domain (NBD1; F508delCFTR) being found at almost 85% of CFTR alleles (http://www.genet.sickkids.on.ca/cftr)^4^. CFTR dysfunction leads to reduced airway surface fluid volume and mucociliary clearance, recurring bacterial infections, chronic inflammation, and the gradual lung function decline that is the main cause of morbidity and mortality^5^.

The F508del mutation causes CFTR misfolding, retention by the ER quality control (ERQC) mechanism, and degradation by the 26 s proteasome^6–8^. Low temperature partially restores F508delCFTR trafficking *in vitro* and the rescued mutant retains channel activity, albeit with lower open probability compared to wild-type CFTR^9^. There has been an intensive effort to find molecules that correct the folding defect and restore trafficking of the mutant^10,11^. In principle there are two general mechanisms by which these corrector compounds could restore F508delCFTR function. Correctors that facilitate its folding by directly binding to the mutant are called pharmacological chaperones while those that modulate the cellular protein environment responsible for folding, trafficking and degradation correctors are referred to as proteostasis regulators^12^.

To date, only two correctors have been approved by the U.S. Food and Drug administration (FDA); VX-809 and its analogue VX-661 (Lumacaftor and Tezacaftor respectively)^13–17^. These drugs provide modest clinical benefit to a subset of patients and there is relatively small improvement in lung function^17,18^. When measured as forced expiratory volume in 1 sec (FEV1), the increase with VX-809 is 2.3–4.4% of the predicted FEV1^19^. This provides a strong rationale for the use of more than one corrector, and tests with combinations of correctors have confirmed that additive effects are achievable. For example, exposing F508del-CFTR expressing cells to both VX-809 and Corr4a *in vitro* gives higher functional correction than either compound alone, evidence that they act at multiple sites, either simultaneously or sequentially along the folding pathway^20^. Other groups have undertaken screens with a view to finding compounds that rescue either additively or synergistically with VX-809 some such as D-01 an aminothiazole do not give correction on their own but do double the level of VX-809 correction in concert with VX-809^21^. Other groups have tested collections of known compounds to search for synergism between correctors^22,23^.

Most correctors have been developed from hits initially obtained by cell-based high throughput screening (HTS) and followed by many iterations of medicinal chemistry and assay of the analogs^10,24,25^. Some cell-based assays measure the trafficking of tagged F508del-CFTR to the cell surface directly^10^ whereas others monitor CFTR function at the plasma membrane as anion conductance or halide flux^24^. In previous work we screened for novel pharmacological chaperones that act synergistically with VX-809 and identified RDR1, a compound which appears to bind to CFTR and provides correction that is additive with that of VX-809^26^. With regard to VX-809 it has been shown to bind to CFTR although its precise site of action on the CFTR molecule is as yet unclear with the NBD1, the transmembrane domain 1 (TMD1) and the interface of the ICL and NBD1 all being suggested as possible sites of action^27–29^. We have now selected eight chemically distinct hit compounds from the original screen and assessed their potential for direct interaction with CFTR in combination with both VX-809 and RDR1. For this we introduce a modified cellular thermal stability assay (CETSA) assay^30,31^ which exploits the ability of ligands to stabilize proteins and increase their solubility. We validate this method for identifying pharmacological chaperones that bind to CFTR and use it to show the possibility of map three functional sites of action. We show that correction provided by multiple pharmacological chaperones can be additive when tested in combination. One triple combination rescued F508delCFTR function >2-fold compared to VX-809 alone when studied using well-differentiated CF bronchial epithelial cells. Since the third site was identified using a molecule obtained directly by HTS, medicinal chemistry is expected to yield analogs that further increase correction. Regardless, our results point to the possibility that VX-809 and at least 2 other pharmacological chaperones bind on NBD1 in CFTR and that these target sites may be targeted to produce synergistic rescue.

## Materials and Methods

### Reagents

Compounds were purchased from commercial sources as follows: VX-809 and VX-661 (S1565 and S7059, Selleckchem Houston, Texas, USA), RDR1 (STK001879 Vitas-M laboratory Champaign, IL. USA), Glafenine (G6895 Sigma-Aldrich St. Louis, MO USA), Domperidone (D8910 Sigma-Aldrich St. Louis, MO. USA), KM11067 (Ryan Scientific Inc. USA) MCG1516A (STK370345 Vitas-M laboratory Champaign IL, USA), 5874896, 5817183, 5359709 and 5476294 (ChemBridge Corp. USA). See Suppl. Table 1. Unless otherwise stated, all compounds were used at 10 μM except VX-809, which was used at 1 μM. VX-809 was used at 1 µM after extensive testing in which it was found to be optimal and not significantly different to the published optimal concentration^11^ (Suppl. Fig. 6).

### CFTR constructs

The CFTR deletion mutations referred to in the text were kindly provided by Prof. Gergely Lukacs (McGill University, Canada)^32^. They were expressed in BHK cells using the pNUT vector and had a triple hemagglutinin tag positioned in the 4^th^ extracellular loop at position 923 of the CFTR cDNA coding sequence. The NBD1 (389–673) plasmid was a human F508del-NBD1 expressed in pET^26^. Stabilized human NBD2 was kindly provided by Prof. Julie Forman-Kay (Hospital for Sick Children, Toronto) and expressed from a pET-SUMO-NBD2 SOL7 plasmid (1193–1445, Q1280E/H1402A/L1436D/Q1411D/Y1307N/S1255L/S1359A)^27^.

### CFTR surface expression assay (CSEA) (HTS -also known as high throughput screening assay)

The CSEA (HTS) assay was performed as before^10,33^. Briefly, F508del-CFTR bearing three tandem hemagglutinin-epitopes (3HA) in the fourth extracellular loop was stably expressed in baby hamster kidney (BHK) cells, plated in 96-well plates, and treated with test compounds for 24 h. Cells were then fixed, and immunostained using a mouse monoclonal anti-HA antibody (Sigma-Aldrich, St. Louis, MO) for quantification of surface CFTR. Hits were those compounds that lacked intrinsic fluorescence and that gave signals that were consistently three standard deviations higher than untreated control cells.

### Antibodies

Anti-CFTR antibodies targeting the N-terminus (sc-10747; anti-CFTR Santa Cruz Biotechnology, Dallas TX) used at 0.5 µg/ml, NBD1 (MAB3484; Millipore, Etobicoke, ON Canada) at 1 µg/ml, C-terminus (MAB3480; Millipore, and MAB25031; R&D Systems, Minneapolis, MN) and hemagglutinin tag at 1 µg/ml (H-9658 Sigma St. Louis, MO) were used.

### Immunoblot

CFTR expression in baby hamster kidney (BHK) cells expressing F508del-CFTR or the deletion mutants of F508del-CFTR were assessed by immunoblotting as described elsewhere^34^. Western blots were probed with the monoclonal anti-CFTR antibodies or anti-HA antibody (see above). The relative amount of each CFTR glycoform was estimated by densitometry using the ImageJ program (Rasband, W.S., National Institutes of Health, http://imagej.nih.gov/ij/)^35^.

### Voltage-clamp studies of primary human bronchial epithelial (HBE) cells

Primary human bronchial epithelial cells (HBE) were from the Primary Airway Cell Biobank at McGill University and were isolated from tissues collected from the Centre Hospitalier de l’Université de Montréal with support from CF Canada and the Respiratory Health Network of the Fonds de Recherche du Québec-Santé. Tissues were obtained at transplantation with informed written consent following protocols approved by the Institutional Review Boards of the CHUM and McGill University.

Furthermore, all protocols utilizing said HBE cells conformed to the previously agreed standard operating procedure for the HBEs as agreed to by the above institutional review board (IRB) (IRB review number A08-M70-14B).

HBE cells were seeded onto fibronectin-coated Snapwell inserts (Corning, Tewksbury MA) and the apical medium was removed after 24 hours to establish an air–liquid interface^36^. Transepithelial resistance was monitored using an EVOM epithelial volt-ohmmeter and monolayers were used after 4 weeks when the resistance was 300–400 Ω•cm^2^. HBE monolayers expressing F508del-CFTR were treated on both sides with 0.1% dimethylsulfoxide (negative control) or 10 μM test compound (except VX-809; 1 μM) in optiMEM containing 2% (v/v) fetal bovine serum. Short-circuit current (*I*sc) was measured across monolayers mounted in modified Ussing chambers and was voltage clamped using a VCCMC6 multichannel current-voltage clamp (both Physiologic Instruments San Diego CA.).

Apical membrane Cl^−^ conductance was functionally isolated by permeabilizing the basolateral membrane with 200 μg/ml nystatin and imposing an apical-to-basolateral Cl^−^ gradient. Basolateral solution contained: 1.2 mM NaCl, 115 mM Na-gluconate, 25 mM NaHCO3, 1.2 mM MgCl_2_, 4 mM CaCl_2_, 2.4 mM KH_2_PO_4_, 1.24 mM K_2_HPO_4_, and 10 mM glucose (pH 7.4). Apical solution contained: 115 mM NaCl, 25 mM NaHCO_3_, 1.2 mM MgCl_2_, 1.2 mM CaCl_2_, 2.4 mM KH_2_PO_4_, 1.24 mM K_2_HPO_4_, and 10 mM mannitol (pH 7.4). Apical mannitol replaced glucose to eliminate current mediated by Na^+^-glucose cotransporters. Successful permeabilization of the basolateral membrane was obvious from the reversal of *I*sc under these conditions. Solutions were maintained at 37 °C and continuously stirred by gassing with 95% O_2_/5% CO_2_. Transepithelial voltage was measured and currents passed using agar bridge Ag/AgCl electrodes. Pulses (1 mV amplitude, 1-second duration) were delivered every 90 seconds to monitor resistance and a PowerLab/8SP interface was used for data acquisition. CFTR was activated by adding 10 µM forskolin + 50 µM genistein to the apical bathing solution and resulting Isc was sensitive to CFTRinh-172 [10 µM^37^], confirming that it was mediated by CFTR.

### Cell culture and transfection

Experiments were carried out on baby hamster kidney cells expressing F508del-CFTR with a triple hemagluttinin tag in the fourth extracellular loop^10^. BHK cells were cultured in DMEM F12 media (Wiscent Inc. Canada. 319-075-CL) with 5% fetal bovine serum (Wiscent Inc. Canada. 080150), Penicillin/Streptomycin (Wiscent Inc. Canada. 450-201-EL) and 500 µM methotrexate (Novopharm Inc. Canada. 02099705)^10^. BHK cells were seeded at 8 × 10^6^ cells/flask and transiently transfected with 18 μg of plasmid and 63 µl of Fugene HD overnight as per the manufacturer’s protocol with the cells utilized after 48 hours. In terms of the culturing and handling of human bronchial epithelial cells, our laboratory follows the protocol set out by Fulcher and Randell^38^.

### Surface biotinylation

BHK cells were grown until 80% confluent and then treated with 10 µM MCG1516A or vehicle for 48 h. The cells were washed twice with phosphate buffered saline (PBS) at 4 °C and EZ Link sulfo-S-S-Biotin (0.5 mg/ml; ThermoFisher Scientific, Waltham MA) was added in PBS for 8 min at room temperature then crosslinking was quenched using 50 mM Tris-Cl, pH 8.0. Cells were collected and lysed using RIPA buffer (20 mM Tris-Cl pH 7.5, 150 mM NaCl, 0.1% v/v sodium deoxycholate, 1% v/v Triton X-100, 0.1% v/v sodium dodecyl sulphate (SDS) and protease inhibitors (cat. 16829800, Roche Diagnostics, Indianapolis IN)) on ice for 20 minutes with vortexing. The lysate was centrifuged for 10 min at 10,000 g at 4 °C and the supernatant collected. Streptavidin beads (10 μl, cat. 20349, Thermo Scientific Rockford, IL) were added to the supernatant and incubated for 2 h at 4 °C with rotation. The beads were collected by centrifugation, washed 4 times with RIPA buffer, run on an SDS-PAGE gel, and immunoblotted to determine the surface CFTR amount^39^.

### FLIPR membrane potential assay

A voltage-sensitive assay was used to assay functional correction of F508del-CFTR in BHK cells. Cells were preloaded with a derivative of the voltage sensitive FLIPR fluorescent dye Bis-(1,3-Dibutylbarbituric acid) Trimethine Oxonol [DiBac_4_]) (https://www.google.com/patents/US6420183), which enters the plasma membrane of the cell and is quenched by the addition of a proprietary quencher to the medium. Activation of CFTR depolarizes the plasma membrane and the dye moves to the inner leaflet of the membrane, which relieves the quenching and increases fluorescence. Assays were performed in 96 well plates containing 25,000 cells per well in 100 μl of medium. Cells were incubated with test compound for 24 h, washed with PBS, exposed to 70 μl dye solution that also contained genistein (GST) (50 µM), and incubated at room temperature for 5 minutes. Dye solution was prepared in low chloride buffer containing (mM) 160 sodium gluconate, 4.5 potassium chloride, 2 calcium chloride, 1 magnesium chloride, 10 mM D-glucose and 10 mM HEPES, pH 7.4. After adding GST and mixing gently with pipette the 96 well plate was placed in a plate reader to read fluorescence. Forskolin (10 µM) was then added and the increase in signal was used to calculate the mean initial rate (i.e. Vmax) as a measure of CFTR function^24^. The mean initial rate is shown in the figures for each treatment with appropriate units.

### Cellular thermal stability assay

BHK cells expressing F508del-CFTR were cultured until they reached 90% confluence. They were then harvested and washed twice in PBS, incubated on ice for 10 min, and centrifuged at 1200 g at 4 °C for 10 min to pellet the cells. The cell pellet was then resuspended in lysis buffer (PBS containing 0.4% v/v Triton X-100 and Roche protease inhibitor cocktail) and incubated for 20 min on ice with occasional vortexing. A protocol^31^ was modified to measure protein in the supernatant and pellet. The lysate was then transferred into separate PCR tubes in aliquots of 40 μl, the test compound was added at 1 or 10 µM final concentration depending on the compound and incubated for 5 minutes on ice. Tubes were then placed in a thermocycler and heated to a range of temperatures (33, 38, 43, 47, 52, 57 and 61 °C) for 10 minutes. The lysate was collected into a fresh tube and centrifuged for 10 min at 10,000 g and 4 °C. The supernatant and pellet were both collected and immunoblotted. Thermal shift was indicated by the temperature at which F508del-CFTR appeared in the pellet fraction^40–42^.

### Differential scanning fluorimetry (DSF)

Melting curves were obtained for NBD constructs using an Mx3005P scanning fluorimeter (Stratagene, San Diego, CA)^26,43^. NBD1, F508del-NBD1, or NBD2 (5X Sypro Orange dye (Sigma-Aldrich)) was diluted to a final concentration of 4 µM, and DMSO vehicle or test corrector were added to aliquots of protein solution such that the final DMSO concentration was 1%. The solution was mixed gently and transferred to a 96-well plate in duplicate. The plate was sealed (Bio-Rad), centrifuged to remove bubbles then run in the fluorimeter fitted with a filter for carboxyfluorescein (FAM, 492 nm) in the excitation path and Cy3 (568 nm) or Texas red (610 nm) filters for emission. Results were exported to Excel (Microsoft) and the apparent melting temperatures were calculated by fitting the curves with the Boltzmann equation using XLFit 4 software (IDBS). Fractional unfolding curves were generated with the following equation:

$$({{\rm{F}}}_{{\rm{T}}}-{{\rm{F}}}_{{\rm{m}}{\rm{i}}{\rm{n}}})/({{\rm{F}}}_{{\rm{m}}{\rm{a}}{\rm{x}}}-{{\rm{F}}}_{{\rm{m}}{\rm{i}}{\rm{n}}}),$$

where F_T_ indicates fluorescence at temperature T, F_min_ indicates the minimum fluorescence and F_max_ indicates the maximum fluorescence. Experiments were performed at least three times.

### Statistics

All results are expressed as the mean ± SEM of n observations. The quality of the data sets used in the short circuit current measurements were compared by one-way analysis of variance (ANOVA) or Student’s t-test using GraphPad Prism version 4 to determine if they fell into the normal range of responses. Differences were considered statistically significant when p < 0.05.

## Results

### RDR1 and VX-809 (Lumacaftor) are synergistic correctors of F508del-CFTR trafficking

**Figure 1:**
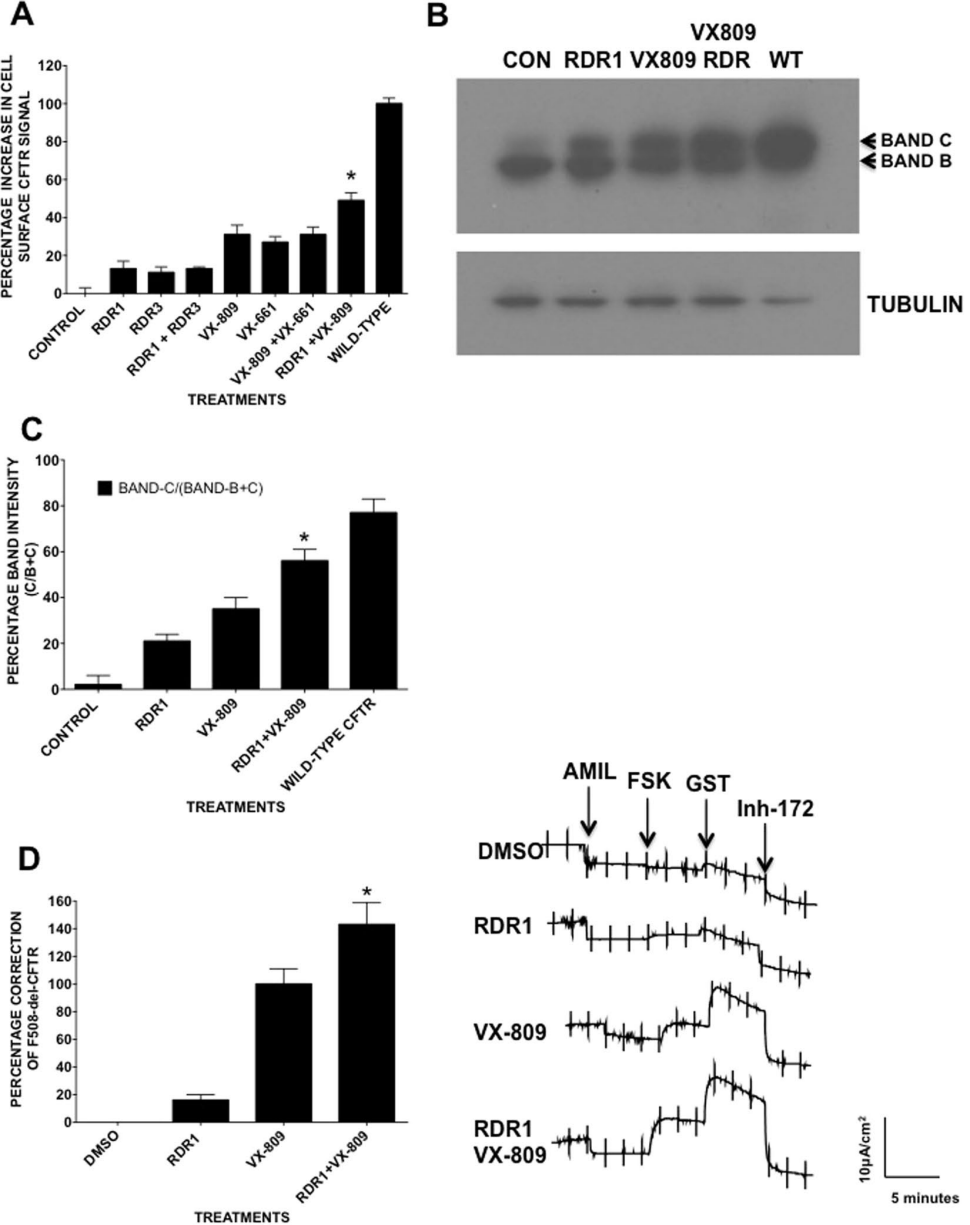
Protein trafficking and electrophysiological assays reveal increased correction of F508del-CFTR by a combination of VX-809 + RDR1. (**A**) Increase in F508del-CFTR protein at the cells surface upon treatment. BHK cells were preincubated with vehicle or RDR1, RDR3 (both 10 μM) and/or VX-661, VX-809 (both 1 μM) for 24 h in the CSEA assay. Results are relative to wild-type surface CFTR (100%). (**B**) Immunoblot of F508del-CFTR in BHK cells after 24-h treatment with RDR1 (10 μM) alone, VX-809 (1 μM) alone, or both and with wild-type CFTR (WT) as a control. (**C**) Relative intensity of bands B and band C in each lane in panel B. (**D**) F508del-CFTR functional expression in well-differentiated primary human bronchial epithelial (HBE) cells determined from the increase in short-circuit current stimulated by acute addition of forskolin + genistein (Δ *I*sc). The basolateral membrane was permeabilized using nystatin and an apical-to-basolateral chloride gradient was imposed. Representative I_sc_ responses of primary HBE cells expressing F508del-CFTR to sequential addition of 10 *µ*M amiloride, 10 *µ*M forskolin, 50 *µ*M genistein, and 10 *µ*M CFTRinh-172 after 24 h preincubation with 0.1% dimethylsulfoxide (vehicle), RDR1 (10 *µ*M) or VX-809 (1 μM) individually and in combination. Data in panels A,C, and D are present as means ± SEM, n = 4, *p < 0.05.

We hypothesized that combinations of different pharmacological chaperones may provide additive or synergistic functional expression approaching that of wild-type CFTR, as reported for combinations of suppressor mutations^20^. To test that hypothesis we evaluated two correctors that had previously been reported to function as pharmacological chaperones, VX-809 and RDR1^24,26,27^, in BHK cells using our cell-based CSEA assay (Fig. 1A). We also tested two analogues of VX-809 and RDR1 that would presumably bind at the same sites as VX-809 and RDR1 and therefore not be synergistic (VX-661 and RDR3, respectively)^11,26^. We confirmed that all these compounds partially correct F508delCFTR when tested individually in this system, with VX-809 being most potent (31% of wild-type functional expression) followed by 27% for VX-661, 14% for RDR1 and 13% for RDR3. The combination of VX-809 + RDR1 gave significantly higher correction (46% of wild type)(as noted by the asterisk). By contrast, VX-809 + VX-661 produced no additivity or synergism and this was also the case with RDR1 + RDR3, indicating that pharmacological chaperones which work at the same site do not have additive effects whereas those which act at distinct binding sites can be synergistic or additive.

To focus on synergism we examined the effects of VX-809 + RDR1. The appearance of mature, complex glycosylated band C protein provides strong evidence that CFTR has escaped ER quality control and trafficked to the Golgi. In addition to giving the strongest signal in the CSEA trafficking assay (>40% increase in surface CFTR according to Fig. 1A), treatment with the combination RDR1 + VX809 also yielded the largest amount of band C on immunoblots (Fig. 1B,C, Suppl. Fig. 1). About 60% of the F508del-CFTR appeared as band C after cells had been treated with the RDR1 + VX-809 combination.

To confirm the presence of surface CFTR detected by the CSEA assay and that the appearance of band C in immunoblots signified the trafficking of CFTR to the cell surface we undertook the use of the surface biotinylation assay (Suppl. Fig. 1A,B). With this approach we were able to demonstrate that all of the corrector treatments but not the control were able to traffick F508del-CFTR to the plasma membrane. Further like Fig. 1A we demonstrated that there is a significant increase in surface CFTR after the treatment with RDR1 and VX-809 (53% band C/band B + C) in combination above the either compound alone or either compound in combination with a chemically related analogues, (VX-809 + VX-661 and RDR1 + RDR3). It should be noted that the parental BHK cells do not express CFTR.

To assess functional correction by the RDR1 + VX-809 combination in airway epithelial cells we measured CFTR-dependent short circuit current (*I*_*SC*_) across CF bronchial epithelial cells after inhibiting sodium current with apical amiloride. Well-differentiated primary HBE cells were preincubated with 10 μM RDR1 and/or 1 μM VX-809 for 24 h, then assayed as the *I*_*sc*_ by acute exposure to forskolin + genistein in modified Ussing chambers (Fig. 1D). Pretreating CF cells with RDR1 + VX-809 produced functional responses that were 40% larger than in cells pretreated with VX-809 alone, indicating rescue equivalent to 14% of the normal non-CF functional response under these conditions. Thus the combination of RDR1 + VX-809 significantly increases F508del-CFTR surface expression compared to either corrector alone.

**Figure 2:**
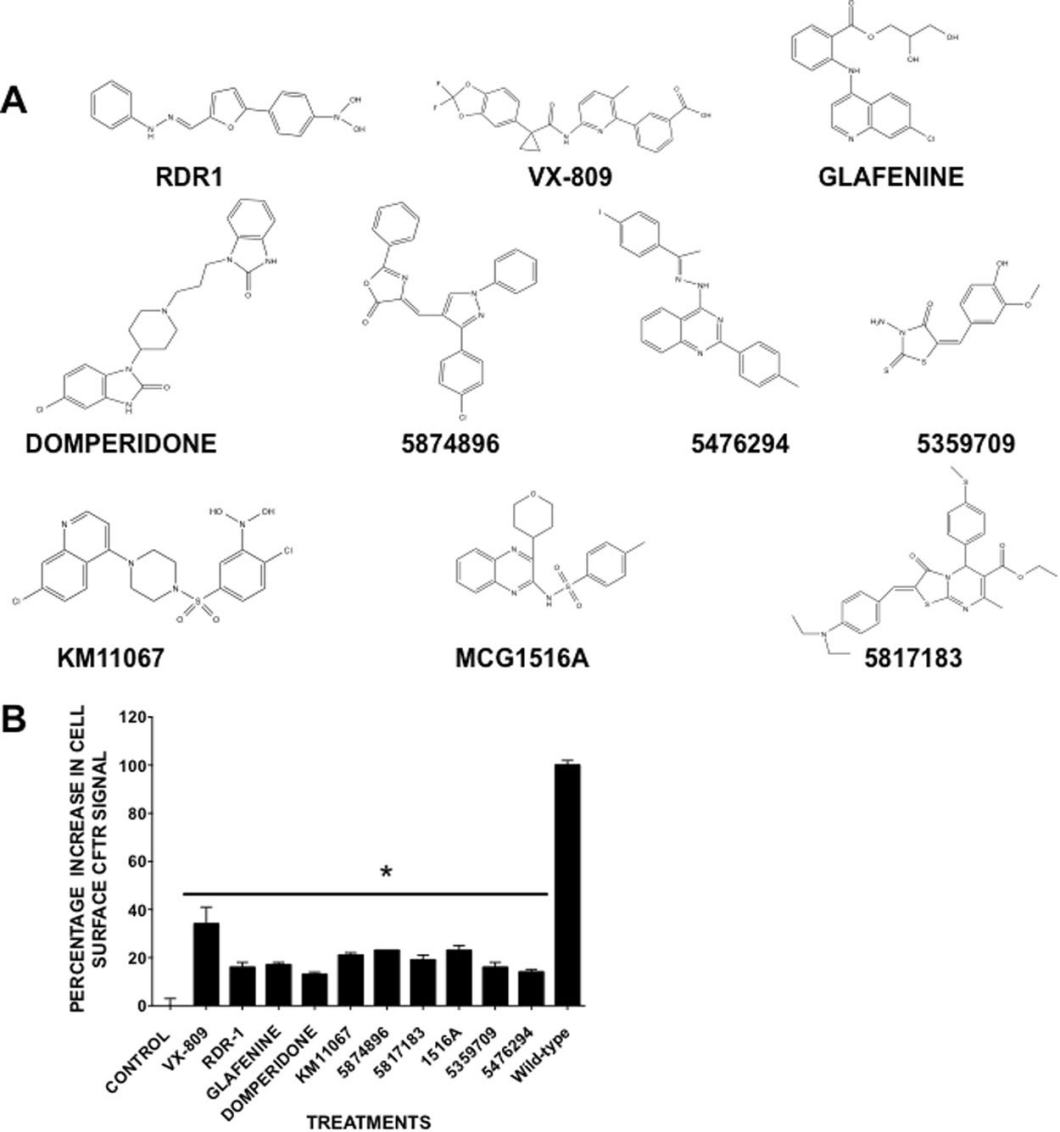
Eight hits obtained by high throughput screening for correctors are confirmed to rescue F508del-CFTR in BHK cells. (**A**) Structures of RDR1, VX-809 and 8 selected hit compounds. (**B**) Cell based CSEA assay measuring surface F508del-CFTR in BHK cells after 24 hour treatment. All compounds were tested at 10 μM except VX-809 (1 μM). Data in panel B is present as means ± SEM, n = 4, *p < 0.05.

We selected a small group of structurally diverse hits from a previous screening campaign to determine if they can also correct F508del-CFTR in a cell-based CSEA trafficking assay (Fig. 2A & Suppl. Table 1)^10,33,34,44^. All eight compounds partially restored F508del-CFTR trafficking, increasing its surface expression by 12–23% after 24 h (Fig. 2B). As expected, these compounds were less efficacious than VX-809 but still gave significant levels of correction comparable to RDR1. To confirm the presence of CFTR on the cell surface after treatment with the designated correctors we also undertook a surface biotinylation assay (Suppl. Fig. 2C,D). This showed that all the correctors tested gave some level of surface CFTR that migrated at the same rate as mature band C. While the compounds did not give the same level of surface CFTR as wild-type CFTR expressing cells or cells treated with VX-809, they did all give amounts of surface CFTR significantly above the control cells.

### MCG1516A is a pharmacological chaperone for F508del-CFTR

**Figure 3:**
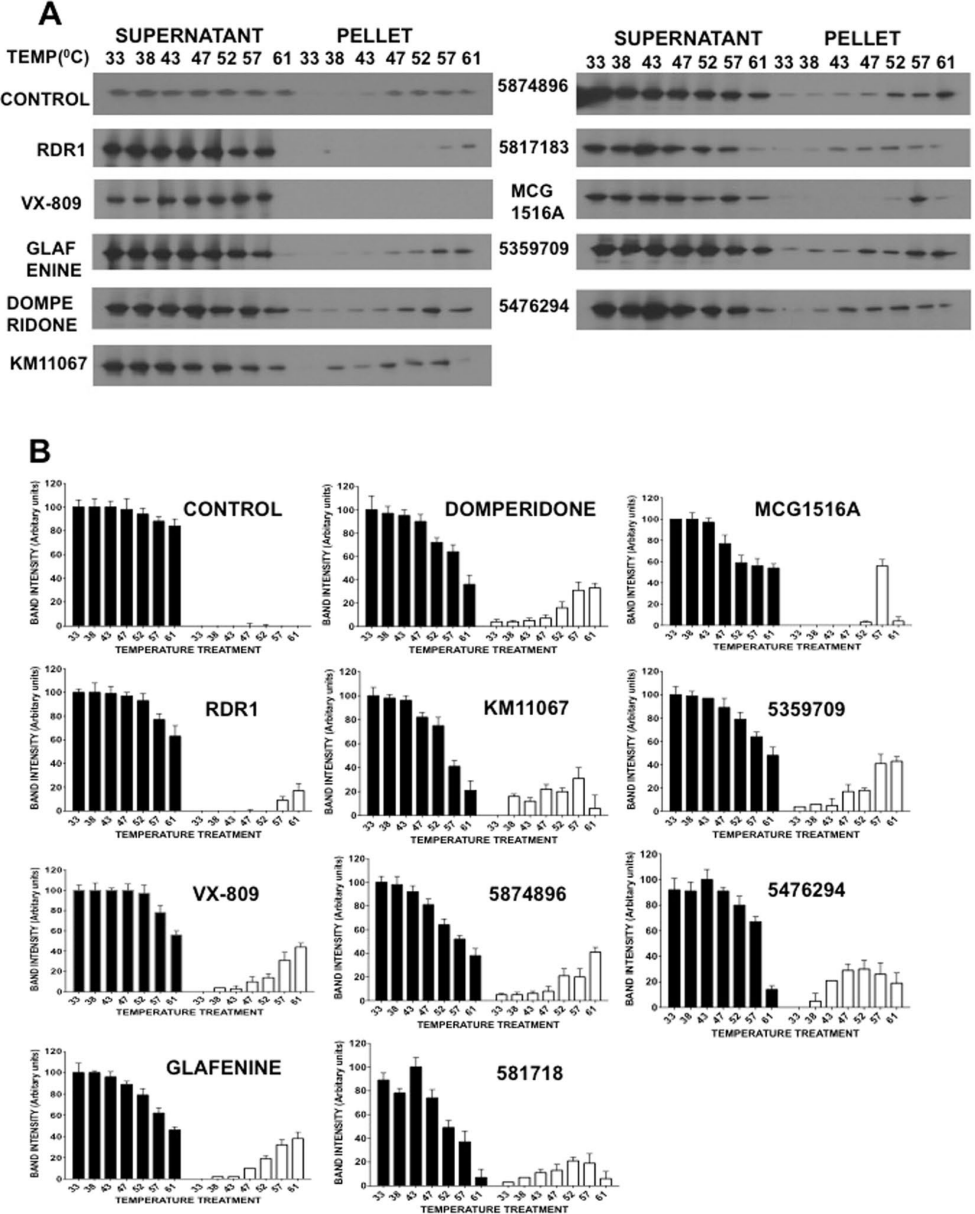
Cellular thermal stability assay (CETSA) to test the binding of correctors to F508del-CFTR. (**A**) Representative immunoblots of the supernatants and pellets after cell lysates are incubated for 10 minutes at different temperatures (33, 38, 43, 47, 52, 57 and 61 °C) in the presence of putative pharmacological chaperones. All correctors were tested at 10 μM except VX-809 (1 μM). Blots were probed with a monoclonal anti-CFTR antibody. (**B**) Graphical representation of the results shown in part A.

To study if correctors identified by CSEA are pharmacological chaperones or not, we first turned to the use of a cellular thermal shift assay (CETSA) (Fig. 3 & Suppl. Fig. 3). BHK cell lysates containing F508del-CFTR were divided into identical aliquots and each was treated for 10 min with 10 μM compound at a different temperature between 33 °C and 61 °C. Lysates were then centrifuged to remove aggregates and the pellets and supernatants were run on polyacrylamide gels and immunoblotted for F508del-CFTR. The concept behind this assay is that as a protein is heated it loses its fold due to the disruption of chemical bonds between amino acids. This results in the protein becoming increasingly misfolded, aggregating and falling out of solution, hence then appearing in the pellet fraction. Compounds that can delay this aggregation increase the protein’s thermostability. Given that the compound is only in contact with CFTR for 10 minutes prior to heating and only in cell lysates would likely preclude any stabilization being the result of proteostasis modulation.

Of the eleven compounds examined the two compounds previously reported to be pharmacological chaperones (RDR1 and VX-809) both show thermal shifts, with RDR1 showing a shift of ~14 °C and VX-809 causing a shift of 18 °C, appearing to remain soluble even at 61 °C this was the largest response detected. Of the remaining compounds, MCG1516A had the largest effect on protein stability. F508del-CFTR first appeared in the pellet at 52 °C in the presence of MCG1516A, much higher than the 38 °C at which F508del-CFTR precipitated in the absence of corrector. By contrast, correctors thought to be proteostasis modulators such as glafenine and KM11067 did not display thermal shifts. Several CFTR correctors of unknown mechanism such as domperidone, 5874896, 5817183 and 5476294 also showed no significant temperature shift in the appearance of CFTR in the pellet. Interestingly, corrector 5359709 shifted the appearance of CFTR in the pellet to a temperature that was 3–5 degrees lower than the control, suggesting its interaction may destabilize CFTR. Destabilization by a CFTR modulator has been reported previously^27^, but how it might improve the surface expression of F508del-CFTR is unclear. In summary, the results suggest that CETSA provides a straightforward and initial tool for identifying correctors that may act as pharmacological chaperones. This data also provides first evidence that MCG1516A may bind to F508del-CFTR therefore we decided to concentrate on MCG1516 in subsequent experiments.

### The dynamics of MCG1516A correction

**Figure 4:**
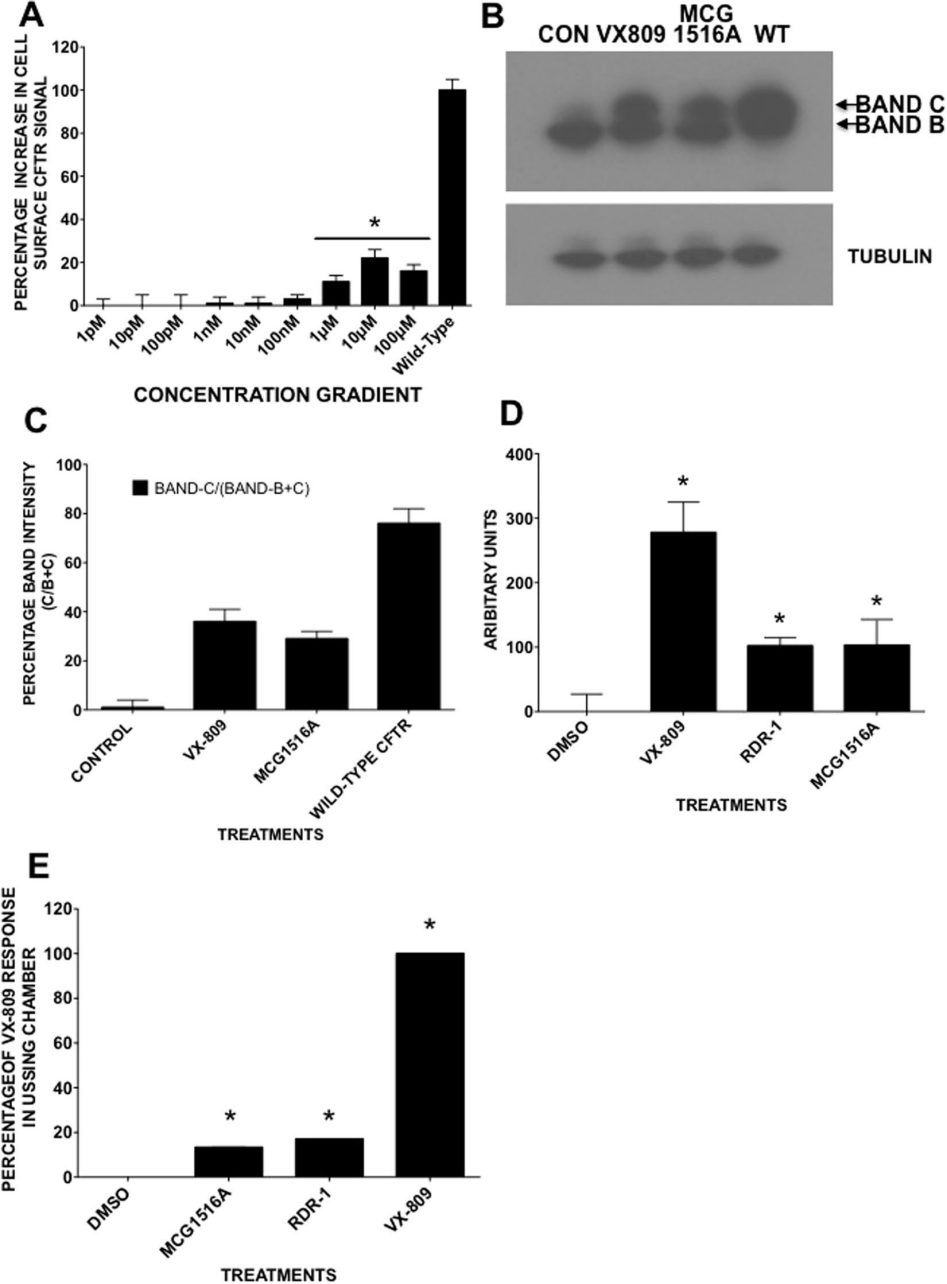
Protein trafficking assays, immunoblotting, FLIPR membrane potential (FMP) assays and electrophysiology demonstrate the rescue of F508del-CFTR. (**A**) Graph of concentration dependence of correction in BHK cells by MCG1516A after 24 h incubation. (**B**) Immunoblot of F508del-CFTR in BHK cells after 24 h treatment with MCG1516A (10 μM) VX-809 (1 μM) and wild-type CFTR (WT). (**C**) Relative band intensities for bands B and C in each lane in panel B. (**D**) FMP assay (FMP) that monitors membrane depolarization induced by forskolin + genistein when cells are pretreated for 24 h with VX-809 (1 μM), RDR1 (10 μM) or MCG1516A (10 μM). (**E**) F508del-CFTR functional expression was assayed as Δ I_sc_ when well differentiated HBE cells are stimulated with forksolin + genistein after 24 h pretreatment with pharmacological chaperones. The basolateral membrane was permeabilized using nystatin and an apical-to-basolateral chloride gradient was imposed. Panels A,C,E, and F show means ± SEM, n = 4, *p < 0.05.

To assess the potency of MCG1516A as a corrector we incubated cells with different concentrations for 24 h and used the CSEA assay to monitor F508del-CFTR rescue (Fig. 4A). The apparent EC_50_ was 930 nM and maximal correction was obtained with 10 μM. Next we measured F508del-CFTR correction by immunoblotting BHK cell lysates containing F508del-CFTR. We found that pretreatment with 10 μM MCG1516A for 48 h produced fully glycosylated CFTR indicating trafficking correction (Fig. 4B, Suppl. Fig. 1B). The correction level was significant, with 32% of the F508del-CFTR protein appearing as band C (Fig. 4C). This data supported by the earlier surface biotinylation result (Suppl. Fig. 2C,D) and establishes that the F508del-CFTR corrected by MCG1516A reaches the plasma membrane of BHK cells after pretreatment with 10 μM MCG1516A for 24 h. Exposure to MCG1516A revealed complex glycosylated CFTR (band C) on the cell surface whereas no F508del-CFTR is detected on the surface of control cells.

### F508del-CFTR that is rescued by MCG1516A is functional in BHK and primary HBE cells

To determine if F508del-CFTR correction by MCG1516A in BHK cells restores anion conductance, we used a FLIPR Membrane Potential (FMP) assay to measure the depolarization that results when CFTR channels at the cell surface are activated in low-Cl^-^ solution (Fig. 4D). We measured the F508del-CFTR response after incubation with 1 μM VX-809, 10 μM RDR1, or 10 μM MCG1516A individually for 24 h. Each treatment caused a significant increase in the response to forskolin + genistein, indicating that functional F508del-CFTR had been rescued. VX-809 exposure produced the largest signal as expected while RDR1 and MCG1516A each gave about one third of the VX-809 response. Responses were abolished by CFTR_inh_-172, confirming that the FMP responses were mediated by F508del-CFTR (data not shown). We also monitored forskolin-stimulated short circuit current (*I*_SC_) across well-differentiated primary HBE cells (Fig. 4E). Exposure to MCG1516A alone increased the forskolin + genistein response to 13.5% of the VX-809 response. Thus MCG1516A is a corrector that restores functional expression in well-differentiated HBE cells from CF patients homozygous for F508del-CFTR.

### MCG1516A, RDR1 and VX-809 all appear to interact with NBD1

**Figure 5:**
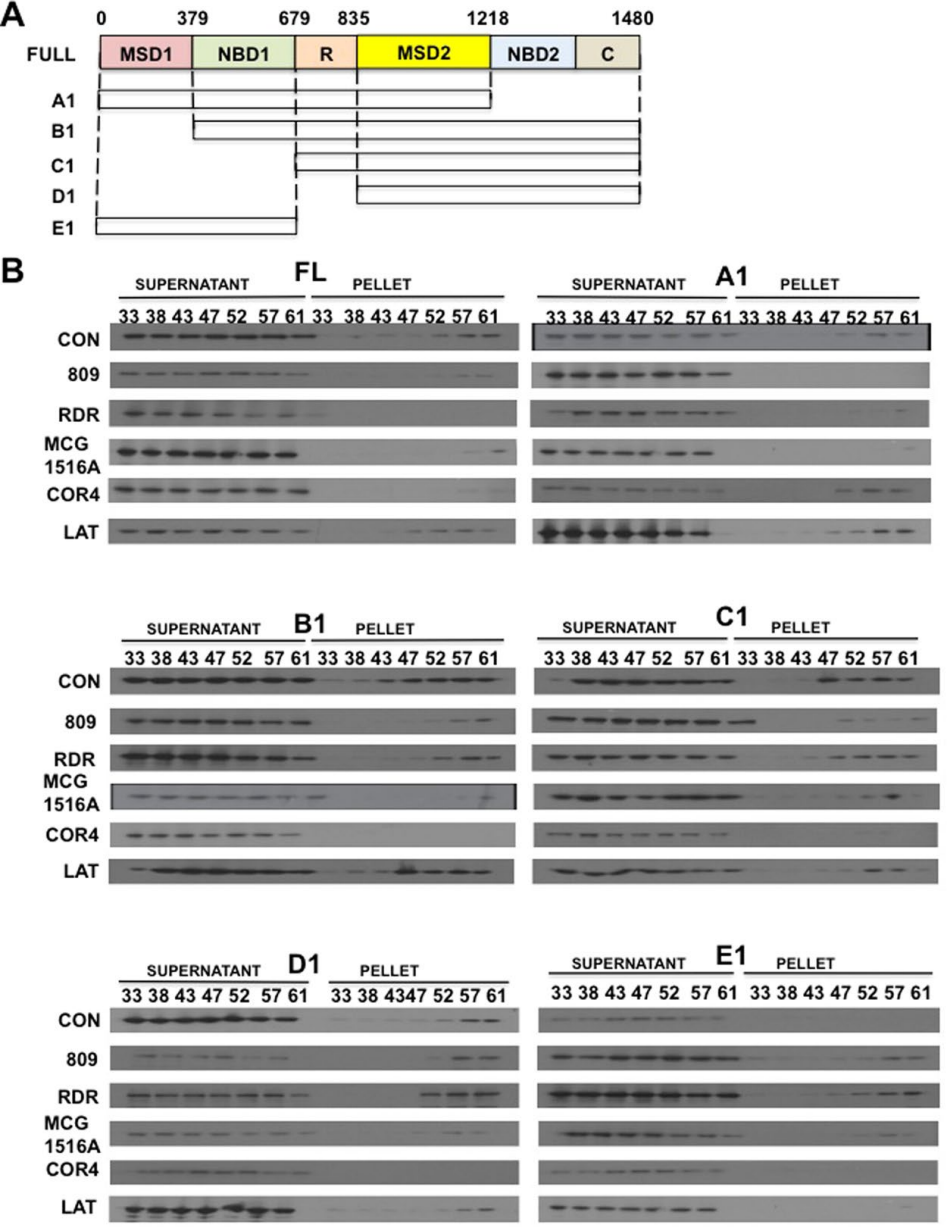
CETSA with deletion mutations identify the domain of F508del-CFTR where pharmacological chaperones act. (**A**) Schematic of full length CFTR (1-1480aa) and 5 deletion mutants used to localize corrector sites of action: A1 (1-1218aa), B1 (389-1480aa), C1 (679-1480aa), D1 (835-1480aa), and E1 (1-694aa). All constructs were HA tagged and expressed in BHK cells using the pNUT vector. (**B**) Immunoblots of supernatants and pellets from after lysates are incubated at different temperatures (33, 38, 43, 47, 52, 57 and 61) in the presence of the RDR1, MCG1516A or latonduine (each at 10 μM) or VX-809 (1 μM). Blots were probed with anti-HA antibody. (For graphical representation see Suppl. Fig. 7).

**Figure 6:**
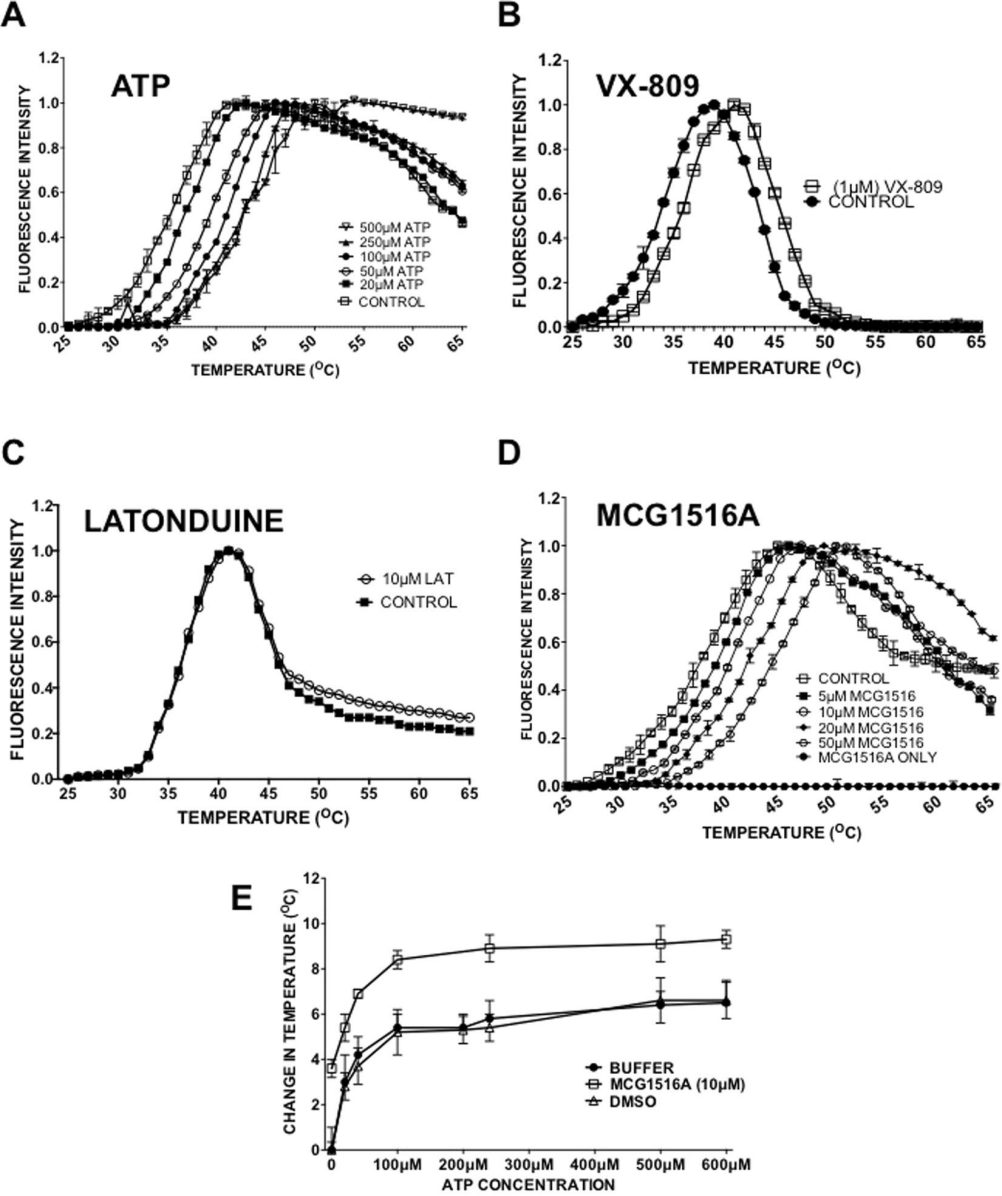
Differential scanning fluorimetry indicates that MCG1516A can interact directly with NBD1. (**A**) Representative melting curves for human NBD1 determined in the presence of increasing ATP concentrations. (**B**) Representative F508del-NBD1 melting curve for human NBD-1 in the presence of VX-809 (1 µM) (**C**) Unfolding of F508del-NBD1 is unaffected by the presence of latonduine. (**D**) Representative F508del-NBD1 melting curve in the presence of MCG1516A. Also shown is the signal obtained with MCG1516A and Sypro orange dye alone (no protein). (**E**) Effect of buffer, 1% DMSO or 10 μM MCG1516A on F508del-CFTR stability in the presence of increasing amounts of ATP. In all parts of the figure the error bars indicate SEM (with an n = 3).

Considering that MCG1516A, RDR1 and VX-809 are all pharmacological chaperones and that RDR1 and VX-809 have both been reported to bind NBD1 although with regard to VX-809 this is still a matter of debate^26–29^, we investigated whether MCG1516A interacts at a third site. To address this we began by using CETSA to analyze a set of F508del-CFTR deletion mutants (Fig. 5 & Suppl. Figs 4, 8, 9 & Suppl. Table 2). The corrector latonduine (10 μM), which acts as a proteostasis modulator by binding to PARP3 and PARP16^25^, served as the negative control in CETSA assays^25,45^. The CFTR corrector corr-4a (COR4a; Cystic Fibrosis Therapeutics lab. (CFFT) corrector compound 4) was also used as a control^46^. It reportedly binds to CFTR via the NBD-2^20^ and evidence to support this is found in Figs 5 and 6 (and Suppl. Figs 8, 9). It should be noted however that this COR4a binding is not specific to CFTR as it has been reported to correct other misfolded proteins^47^. All the prospective pharmacological chaperone-type correctors altered the thermostability of full length F508del-CFTR. The F508del-CFTR deletion mutant A1 was stabilized by all correctors except COR4a, consistent with its interaction at NBD2. There was a clear thermal shift with the MSD1 deletion mutation B1 after treatment with RDR1, VX-809 MCG1516 A and COR4a which was absent with the proteostasis modulator control latonduine, however it was variable between the three correctors. The shift was largest with MCG1516A and COR4a (14 °C) followed by RDR1 (7 °C) and VX-809 (3 °C). The smaller shift may indicate a weaker interaction when MSD1 is absent. Although the three correctors did not cause thermal shifts for the MSD1-NBD1 mutant C1 or the MSD1-NBD1-R-domain mutant D1, the mutant E1 comprising only MSD1-NBD1 was destabilized by all three correctors indicating they interact directly with this fragment. Interestingly, the correctors caused more protein instability as manifest by their appearance in the pellet at various temperatures. This suggests a role for the R domain and MSD2 in F508del-CFTR folding correction. The results are consistent with the idea that all three putative pharmacological chaperone-type correctors interact with NBD1 although stabilization by VX-809 is stronger in the presence of MSD1, indicating MSD1 may play a role in the folding induced by VX-809.

To further support that the concept that MCG1516A may bind to NBD1 we examined the stability of purified human F508del-NBD1 *in vitro* using differential scanning fluorimetry (DSF; Fig. 6). We used different concentrations of ATP (Fig. 6A) as a positive control since ATP binding at the interface between the NBDs is well established^26,48^. We found a concentration-dependent increase in the thermal stability of F508del-NBD1 down to 20 µM ATP, the lowest concentration tested. We also showed that VX-809 a compound reported to interact with NBD-1 increased the thermal stability of NBD-1 (Fig. 6B). We then assayed MCG1516A over the range 5 μM–50 µM and again observed a concentration-dependent increase in thermal stability (Fig. 6D). The compound alone did not produce detectable fluorescence in the presence of Sypro orange at any temperature tested. Moreover latonduine, a proteostasis modulator-type corrector that does not bind F508del-CFTR did not cause any thermal shift in the CFTR NBD1 as expected (Fig. 6C). It should be noted that the F508del-CFTR thermal stabilization with 5 μM MCG1516 A was similar to that produced by 20 μM ATP (compare Fig. 6A,D). To test if MCG1516A stabilizes F508del-NBD1 by interacting with the ATP binding sites we examined whether MCG1516A competes with ATP for thermal stabilization of F508del-NBD1. A range of ATP concentrations were added to F508del-NBD1 in the presence or absence of 10 µM MCG1516A and thermal stability was assessed (Fig. 6E). The results show that MCG1516A in the presence of ATP stabilizes F508del-NBD1 by an additional 3 °C compared with ATP alone, suggesting it acts at a site that is distinct from the nucleotide binding fold (Fig. 6E).

Finally, we confirmed using DSF that MCG1516A, like RDR1 and VX-809, does not affect the melting temperature of NBD2 (Suppl. Fig. 5). However, COR4a did cause an (2 °C) increase in thermostability of purified NBD2 consistent with the idea of COR4a interacting with this domain^20^. These data indicate that MCG1516A potentially binds F508del-NBD1, albeit at a different site than ATP.

### MCG1516A acts synergistically with RDR1 and VX-809 to correct F508del-CFTR trafficking in BHK cells

**Figure 7:**
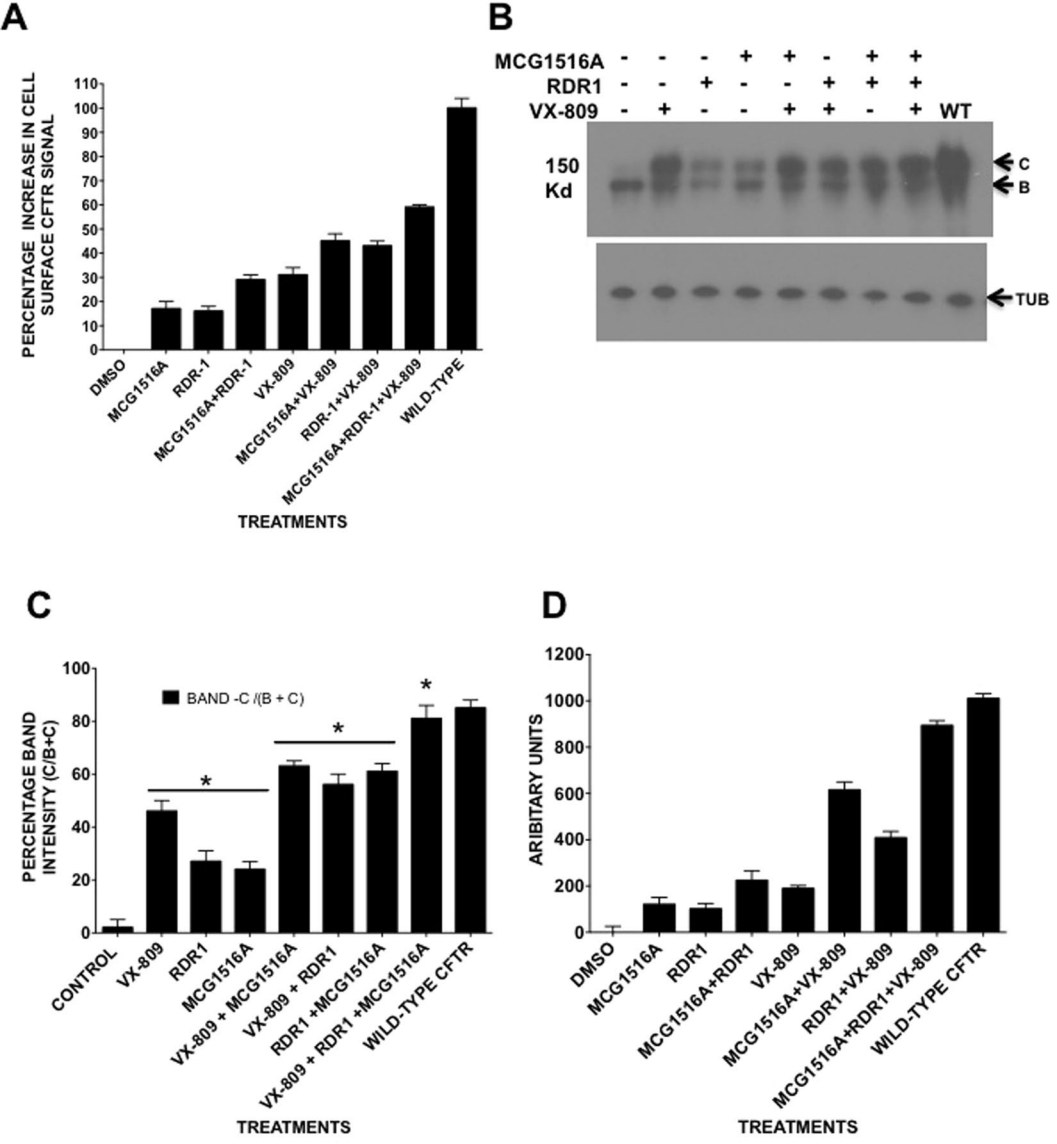
Effects of RDR1, VX-809 and MCG1516A on F508del-CFTR when tested individually and in combination. (**A**) Surface expression of F508del-CFTR with 10 μM MCG1516A (1516a), 10 μM RDR1 and 1 μM VX-809 when pretreated for 24 h separately and combined using the CSEA assay. (**B**) Immunoblot of F508del-CFTR in BHK cells after the same treatments as in A. (**C**) A graph of the relative band intensity of band C when compared to the combined intensities of bands B and C in panel B. (**D**) FLIPR membrane potential assay (FMP) results in which correction by treatments shown in A is measured functionally as the depolarization that results from F508del-CFTR channel activation by genistein + forskolin.

To determine if MCG1516A acts additively or synergistically with other pharmacological chaperones in our cell based CSEA assay (Fig. 7A), it was tested at 10 μM in combination with RDR1 (10 μM) and VX-809 (1 μM) for 24 hours. Under these conditions its effect was additive with the correction produced by RDR1 or VX-809. When tested individually, MCG1516A and RDR1 gave increases of 17% and 16% in surface CFTR signal, respectively. When used together the increase was 29.5%; i.e. approximately additive. Summing the individual effects of MCG1516A and VX-809 predicted a 31% increase in cell surface CFTR, whereas the combination produced a 45% increase. The triple combination of RDR1 + VX-809 + MCG1516A increased correction of F508del-CFTR by 59%.

To confirm the additive-synergistic effects of these correctors on surface expression, we measured the amount of band C F508del-CFTR by immunoblotting after treatment with various combinations of MCG1516A, RDR1 and VX-809 (Fig. 7B,C, Suppl. Fig. 1C). A similar pattern was obtained, i.e. more mature band C relative to band B, with the combination of VX-809 and RDR1 compared to individual correctors. A triple combination produced the highest ratio of band C to band B, with 90% in the band C form. This ratio is similar to that for wild-type CFTR expressed in BHK and CFBE41o- cells (data not shown) and may represent an upper limit for correction under these conditions. The small differences one sees between the ratios of surface CFTR detected in the CSEA assay (Fig. 7A) and the mature band C seen in the immunoblot (Fig. 7B) is most likely due to the amount of CFTR being trafficked from the Golgi apparatus to the plasma membrane and the amount retained in vesicles beneath the cell surface.

To determine if increased surface expression resulted in higher CFTR channel activity at the plasma membrane we measured functional correction using the FMP assay after pretreatment with the same corrector concentrations for 24 h (Fig. 7D). MCG1516A was synergistic with both RDR1 and VX-809. Individually, MCG1516A and RDR1 gave mean responses of 109 and 97 units, respectively, whereas pretreatment with the combination produced a response of 230 units. The combination of VX-809 with MCG1516A was even more synergistic, increasing the functional response from 211 units with VX-809 alone to 615 units in combination with MCG1516A. The triple combination yielded the highest functional expression, with 894 units which was approximately 90% of the wild-type response. These results show that the increases in surface expression and function induced in BHK cells by MCG1516A are additive with those induced by RDR1 and VX-809.

### Correction by MCG1516A is additive with that by RDR1 and VX-809 in polarized HBE cells from CF patients

**Figure 8:**
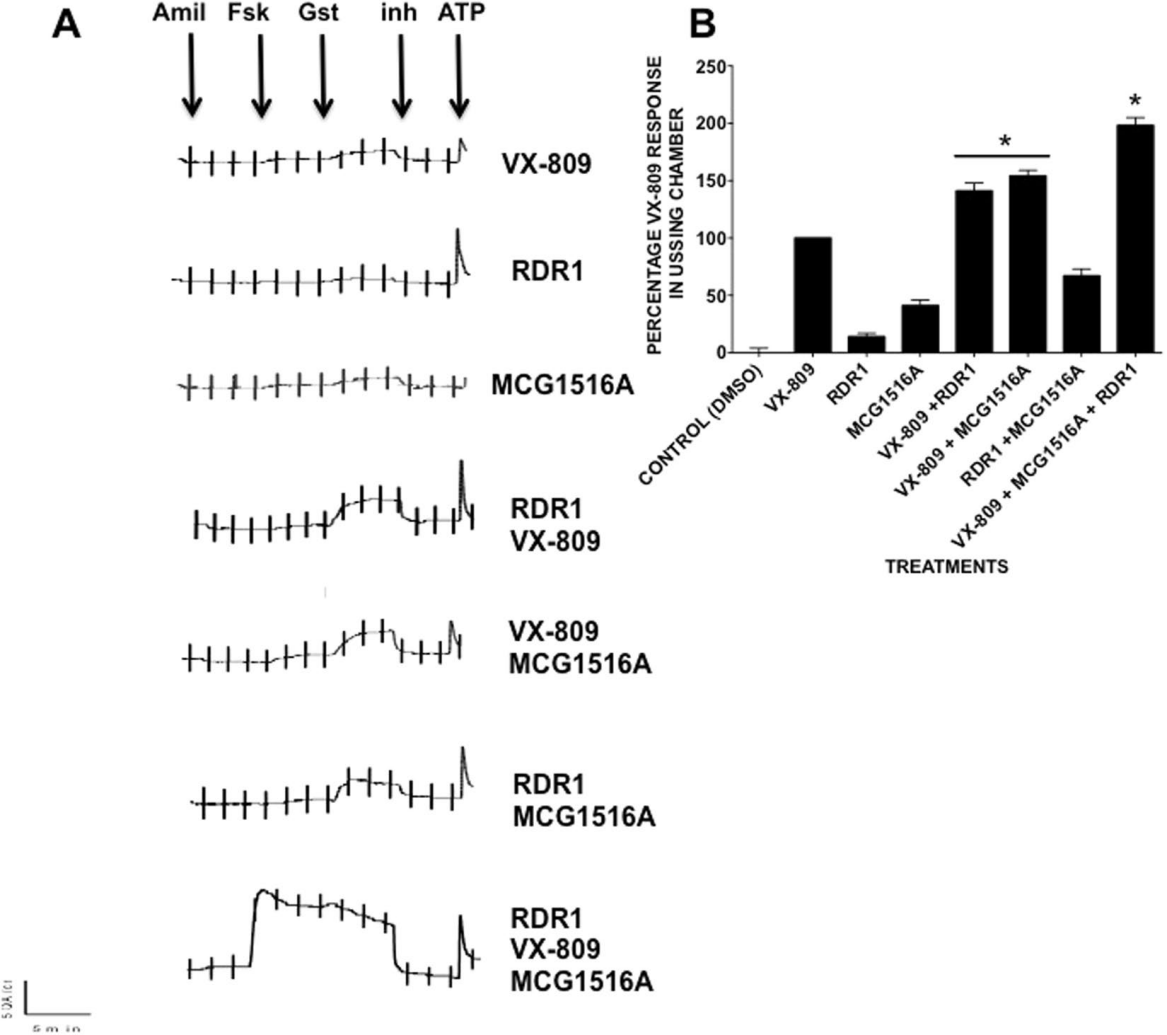
Increased correction by the triple combination MCG1516A + RDR1 + VX-809 in primary HBE cells. Functional expression of F508del-CFTR in well differentiated primary HBE cells. The basolateral membrane was permeabilized using nystatin and an apical-to-basolateral chloride gradient was imposed. (**A**) Representative *I*sc responses to 10-*µ*M forskolin, 50 µM genistein, and 10 *µ*M CFTRinh-172 after 24 hours of exposure of F508del-CFTR HBE cells to 0.1% dimethylsulfoxide (vehicle), MCG1516A (10 μM), RDR1 (10 *µ*M) VX-809 (1 μM) and the all combinations of the three. (**B**) Summary of short-circuit current responses (ΔI_sc_) to acute forskolin + genistein exposure relative to VX-809 alone in the same experiment. Means ± SEM, n = 3_independent experiments, *p < 0.05.

Correctors that are efficacious in cell lines often do not work in more physiologically relevant cell models^49^. We therefore examined functional correction using well-differentiated primary HBE cultures cultured at the air-liquid interface and mounted in Ussing chambers. Cell were pretreated with individual correctors or with different corrector combinations (Fig. 8)^50^. We used VX-809 as a benchmark and its response was set at 100% for comparison purposes. I_sc_ responses confirmed that each compound provides some functional correction when tested alone. MCG1516A caused rescue equivalent to ~42% of the VX-809 level while RDR1 correction was much less, ~13% that of VX-809 (Fig. 7). The combination of RDR1 + VX-809 gave 144% of the VX-809 signal whereas the triple combination that also included MCG1516A gave 196% of the VX-809 functional correction. These data are consistent with the BHK results and point to the triple combination of correctors in combination with a potentiator as a potent cocktail for F508del-CFTR rescue. It should be noted that the triple combination also reached maximal activation upon the addition of forskolin suggesting that in combination the three compounds also may display some corrector ability on top of its corrector ability.

## Discussion

Class 2 mutations such as F508del cause the misfolding, mislocalization (to the ER rather than the plasma membrane) and premature degradation of CFTR. The ability of suppressor mutations to partially restore its biosynthesis suggests that folding is influenced by multiple sites that may be distant from F508^14,15,20,51,52^. F508del-CFTR is kinetically and thermodynamically unstable at 37 °C and studies suggest that NBD1 stabilization reduces F508del-CFTR misfolding^4,53^, although correction (65–80%) is most pronounced when combined with mutations that stabilize the domain-domain interface between NBD1 and MSD2^14,20,54^. This dependence on multiple mutations suggests that interaction of small molecule correctors with at least two sites may be required to achieve optimal correction of F508del-CFTR.

The pharmacology of corrector drugs is in its infancy however development of pharmacological chaperones that rescue ER-retained F508del-CFTR is widely regarded as a promising therapeutic strategy for CF. VX-809 is approved for clinical use in the combination therapy Orkambi and several other correctors are currently in clinical trials^24,55,56^. The correction provided by pharmacological chaperones is generally higher than correctors utilizing other mechanisms^25^, nevertheless the clinical improvement achieved with single correctors has been modest reinforcing the need for more efficacious therapies^16^. This has spurred the search for other novel correctors and for combinations of correctors that together will provide the necessary correction. In his 2014 study Verkman and his collaborators screened 100,000 compounds in combination with VX-809 in search of synergy. D-01 their most promising compound did give synergy in combination with VX-809 but was not a corrector on its own^21^. Interestingly in their work they found that NBD2 needed to be present suggesting a possible site of interaction. Others have explored known corrector compounds such as the CFFT collection of correctors to show that additivity occurs when combinations of compounds are used. Some such as the Cebotaru group used such correctors to test their ability to correct other class 2 CFTR mutations such as G85E and L1077P with varying success. What was interesting in this work was that while C4 and C18 in combination could rescue some class 2 mutations it could not rescue others. This provides further support for the concept of theratyping separating mutations based on their response to correction^23,57^. In their 2014 work the Becq show that using combinations of correctors provides significantly more potent rescue than each compound alone and that if three correctors are used together then the rate of correction reached 82% of the cells tested^22^. In short the combination studies up to now highlight the potential of this approach, the need for novel correctors to address those mutations that current correctors do not rescue and the fact that more than 2 correctors may be necessary to attain the levels of rescue required clinically.

In this study we have shown that three potential pharmacological chaperones can in combination with a potentiator provide synergistic improvement in the functional expression of F508del-CFTR^14,20^. Our evidence suggests that all three correctors appeared to target NBD1, suggesting it bears three functionally distinct sites. The concept that VX-809 binds to NBD1 is not new^27^ however, there is evidence to suggest that VX-809 may bind elsewhere in CFTR such as the MSD1 domain or even in more than one site^28,29,58,59^. Such lack of clarity regarding the VX-809 interact site is due in no small part to the dynamic nature of CFTR protein folding.

We began by testing two correctors (VX-809 and RDR1) that had been reported previously to be pharmacological chaperones. The results indicate that the effects of VX-809 + RDR1 enhances F508del-CFTR correction more than either compound alone and are additive. Well-differentiated HBE cells homozygous for F508del-CFTR reached functional expression that was 140% of the level attained by VX-809 alone. This caused us to wonder if a third corrector might increase the level of F508del-CFTR rescue even further. To date there has only been functional evidence for two corrector sites on CFTR.

To investigate this we selected a set of chemically diverse F508del-CFTR correctors that had been identified in our previous screens and confirmed their ability to rescue F508del-CFTR when tested individually. The strongest hits were 5874896 and MCG1516A, which each produced a ~23% increase in surface CFTR signal. This was equivalent to ~70% of the rescue provided by VX-809, and was substantially higher than that provided by RDR1 (16%). We then employed a novel cellular thermal shift assay (CETSA) to determine if these new correctors bind and therefore are likely to act as pharmacological chaperones. Using this approach and differential scanning fluorimetry we generated data that supported the concept that MCG1516A binds to full-length F508del-CFTR and also to recombinant NBD1 but not to NBD2. Although it should be remembered that both of the constructs used are highly engineered in nature. Functional correction was also observed in well-differentiated F508del-CFTR HBE cells, which are generally regarded as the gold standard model for evaluating CFTR modulators (Fig. 4).

How much CFTR correction is required for clinical benefit remains uncertain although 25% of wild-type function has been suggested^60,61^ and CF carriers with 50% CFTR function are almost asymptomatic. To date, the best corrector + potentiator combination (Orkambi™), which restores functional expression to 11–15% that of wild-type CFTR in HBE cells *in vitro*, increases lung function by only 4.2% when measured by FEV1^16^. Ongoing clinical trials of corrector combinations containing two experimental corrector drugs have reported up to 12% improvement in FEV1 (Vertex pharmaceuticals press release. 2017) which, based on the experience of G551D patients receiving Kalydeco should provide considerable clinical benefit. A triple combination could potentially increase this benefit further therefore we explored the possibility of a third pharmacologically significant site using MCG1516A, measuring CFTR rescue *in vitro* after pretreatment with VX-809, RDR1 and MCG1516A individually and in various combinations.

Interestingly, we found that MCG1516A increases F508del-CFTR when used alone, together with either VX-809 or RDR1, and when all three correctors were used (Figs 7 and 8). The triple combination RDR1 + VX-809 + MCG1516A gave a functional response that was 196% of that attained by VX-809 alone in well-differentiated primary HBE cells. If a similar correction were achieved in patients it would be while not necessarily being sufficient to alleviate CF symptoms would be a major step towards such a goal. This is an exciting result in that RDR1 and MCG1516A are both original hit compounds from screens that have undergone no optimization by medicinal chemistry. Hence they may be the first members of new classes of corrector compounds, and progenitors of compounds that may be significantly more effective. As mentioned in the results the maximal level of functional response occurred upon forskolin response. This suggests that one of the compounds may also have potentiator function along with its corrector ability but that this only manifests itself when the three compounds work in concert. For this profile is not present in any of the compounds alone or in any of the two compound combinations tested. This result is intriguing but hard to explain it may be due the three compounds triggering a fold that is more ‘wild-type like’ or a fold that in someway compensates for the well reported gating deficiency of F508del-CFTR^9^.

Developing multiple CFTR modulators targeting distinct sites may make it possible to avoid combinations in which the constituents negatively interact. For example, lumacaftor (VX-809) increases metabolism of ivacaftor (VX-770) necessitating a higher dose of the latter when used in combination as Orkambi. Conversely, ivacaftor reduces F508del-CFTR surface expression in cell cultures incubated with lumacaftor^13,62^ although function is maintained when clinically relevant (low nM) concentrations are used^63^, probably due to cell accumulation of ivacaftor and increased potentiation of the channels that remain. Nevertheless, combinations are expected to be more effective when their components do not negatively interact.

Currently available pharmacological chaperones do not rescue all class 2 CFTR mutants. Thus, VX-809 partially corrects F508del-CFTR but has little if any effect on some class 2 mutants such as N1303K and G85E^64,65^. Combining multiple correctors may increase the rescue of these mutants if, for example, the binding of one modulator overcomes a barrier to folding that limits the action of other correctors in the combination. Identifying their sites of action will clarify CF theratypes and facilitate personalized medicine approaches by predicting which correctors are best suited for individual patients^57^.

We have demonstrated three compounds that all act as pharmacological chaperones for F508del-CFTR using a novel CETSA assay. Each of these correctors worked additively or synergistically with the other two suggesting they act at distinct sites. Results with deletion mutants suggested they interact with NBD1 and this was supported by interaction with a recombinant NBD1 polypeptide, although VX-809 binding was enhanced by the presence of MSD1. These data are consistent with a recent report that VX-809 binds to NBD1^27^ and with previous reports of corrector binding, including RDR1 interaction with NBD1^24,26,27^. The possibility that three different correctors (RDR1, VX-809 and MCG1516A) act synergistically by engaging NBD1 is perhaps not surprising when it is the domain bearing the F508del mutation. However, this does not preclude the possibility that other correctors like Cor4a will be identified which interact at sites elsewhere on CFTR yet can partially rescue F508del-CFTR.

## Electronic supplementary material

Supplementary data
