# Supplementary material for: A novel triple combination of pharmacological chaperones improves F508del-CFTR correction
Source: Sci Rep. 2018 Jul 30;8:11404. doi: 10.1038/s41598-018-29276-y (PMC6065411; doi:10.1038/s41598-018-29276-y)
Supplement: Supplementary file 1 — Supplementary data [file 41598_2018_29276_MOESM1_ESM.pdf]

SUPPLEMENTARY DATA

A novel triple combination of pharmacological chaperones improves F508del-CFTR correction.

Graeme W. Carlile[1]*, Qi Yang[1], Elizabeth Matthes[2], Jie Laio[2], Stevo Radinovic[1,3], Carol Miyamoto[1], Renaud Robert[2], John W. Hanrahan[2], and David Y, Thomas[1]

[1]Cystic Fibrosis Translational Research Center, Dept. of Biochemistry McGill University Montreal Quebec Canada, H3G 1Y6

[2] Cystic Fibrosis Translational Research Center, Dept. of Physiology McGill University Montreal Quebec Canada, H3G 1Y6

[3]National Research Council, Biotechnology Research Institute, 6100 Royalmount Ave, Montreal Quebec Canada, H4P2R2

SUPPLELEMENTARY FIGURE CONTENTS

SUPPLEMENTARY TABLE 1. List of the corrector compounds selected for testing

SUPPLELEMNTARY FIGURE 1. Uncropped versions of western blot autoradiographs that appear in figures 1B, 4B, 7B and their tubulin loading controls.

SUPPLEMENTARY FIGURE 2. Demonstration of F508del-CFTR being trafficked to the cell surface using surface biotinylation assays.

SUPPLEMENTARY FIGURE 3. Uncropped versions of the western blot autoradiographs presented in figure 3 and their tubulin loading controls.

SUPPLEMENTARY FIGURE 4. Demonstration of the expression of F508del-CFTR deletion mutants in BHK cells by immunoblot.

SUPPLEMENTARY FIGURE 5. Thermal stabilization of NBD2 in the presence of correctors.

SUPPLELEMNTARY FIGURE 6. Uncropped versions of western blot autoradiographs that appear in Supplementary figure 2.

SUPPLEMENTARY FIGURE 7. Data included to demonstrate that there is no difference in response between 1μM and 3μM for VX-809 for our assays.

SUPPLEMENTARY FIGURE 8 Graphical representation of the CETSA results presented in Figure 5 and SUPPLEMENTARY TABLE 1.

SUPPLEMENTARY FIGURE 9 Uncropped versions of the western blot autoradiographs that appear in Figure 5 and their tubulin loading controls

SUPPLEMENTARY TABLE 2 Results from Figure 5 presented in table form.

SUPPLEMENTARY TABLE 1

NAME	FULL CHEMICAL NAME	STRUCTURE	Log P
**RDR1**	**5-(4-nitrophenyl)-2-furaldehyde 2-phenylhydrazone**	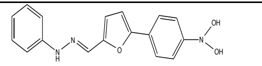	**2.4**
**VX-809 (Lumacaftor)**	**3-(6-(1-(2,2-Difluorobenzo [d] [1,3] dioxol-5-yl) cyclopropanecarboxamido)-3-methylpyridin-2-yl) benzoic acid**	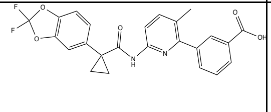	**4.4**
**Glafenine**	**2,3-Dihydroxypropyl 2-((7-chloroquinolin-4-yl)amino)benzoate**	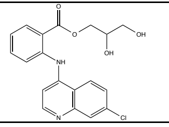	**3.6**
**Domperidone**	**5-Chloro-1-(1-[3-(2-oxo-2,3-dihydro-1H-benzo [d] imidazol-1-yl) propyl] piperidin-4-yl)-1H-benzo [d] imidazol-2(3H)-one**	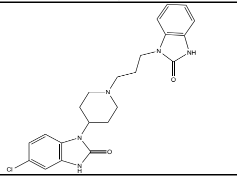	**3.9**
**Km11067**	**7-chloro-4-{4-[(4-chloro-3-nitrophenyl)sulfonyl]piperazino}quinoline**	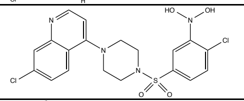	**4.1**
**5874896**	**'4-{[3-(4-chlorophenyl)-1-phenyl-1H-pyrazol-4-yl]methylene}-2-phenyl-1,3-oxazol-5(4H)-one**	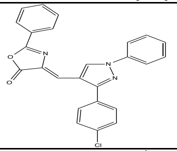	**3.7**
**5817183**	**'ethyl 2-[4-(diethylamino) benzylidene]-7-methyl-5-[4-(methylthio)phenyl]-3-oxo-2,3-dihydro-5H-[1,3] thiazolo [3,2-a]pyrimidine -6-carboxylate**	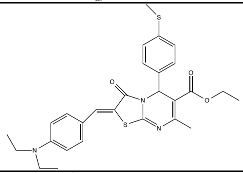	**4.2**
**MCG1516A**	**4-methyl-n-[3-(morpholin-4-yl)quinoxalin-2-yl] benezenesulfonamide**	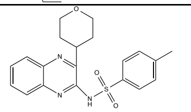	**3.9**
**5359709**	**3-amino5-4-hydroxy-3-methoxybenzylidene)-2-**	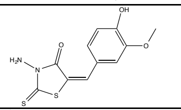	**3.3**
**5476294**	**1-(4-iodophenyl) ethanone [2-(4-methylphenyl)-4-quinazolinylhydrazone**	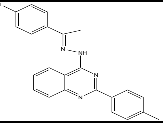	**2.7**
**VX-661**	**1-(2,2-difluoro-1, 3-benzodioxol-5-yl)-N-[1-[(2R)-2,3-dihydroxypropyl]-6-fluoro-2-(1-hydroxy-2-methylpropan-2-yl) indol-5-yl] cyclopropane-1-carboxamide]**	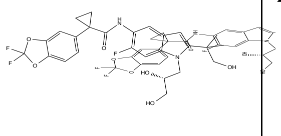	2.9
**RDR3**	**5-(2-((5-(3-bromo-4-nitrophenyl)-2-furyl) methylene) hydrazine)-2-chlorobenzoic acid**	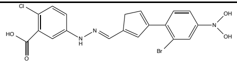	2.6

SUPPLEMENTARY TABLE 1 This is a table of the compounds used in the initial screen and in figure 1. It includes the common name, chemical name (IUPAC name) and 2D structure of each compound.

SUPPLEMENTARY FIGURE 1

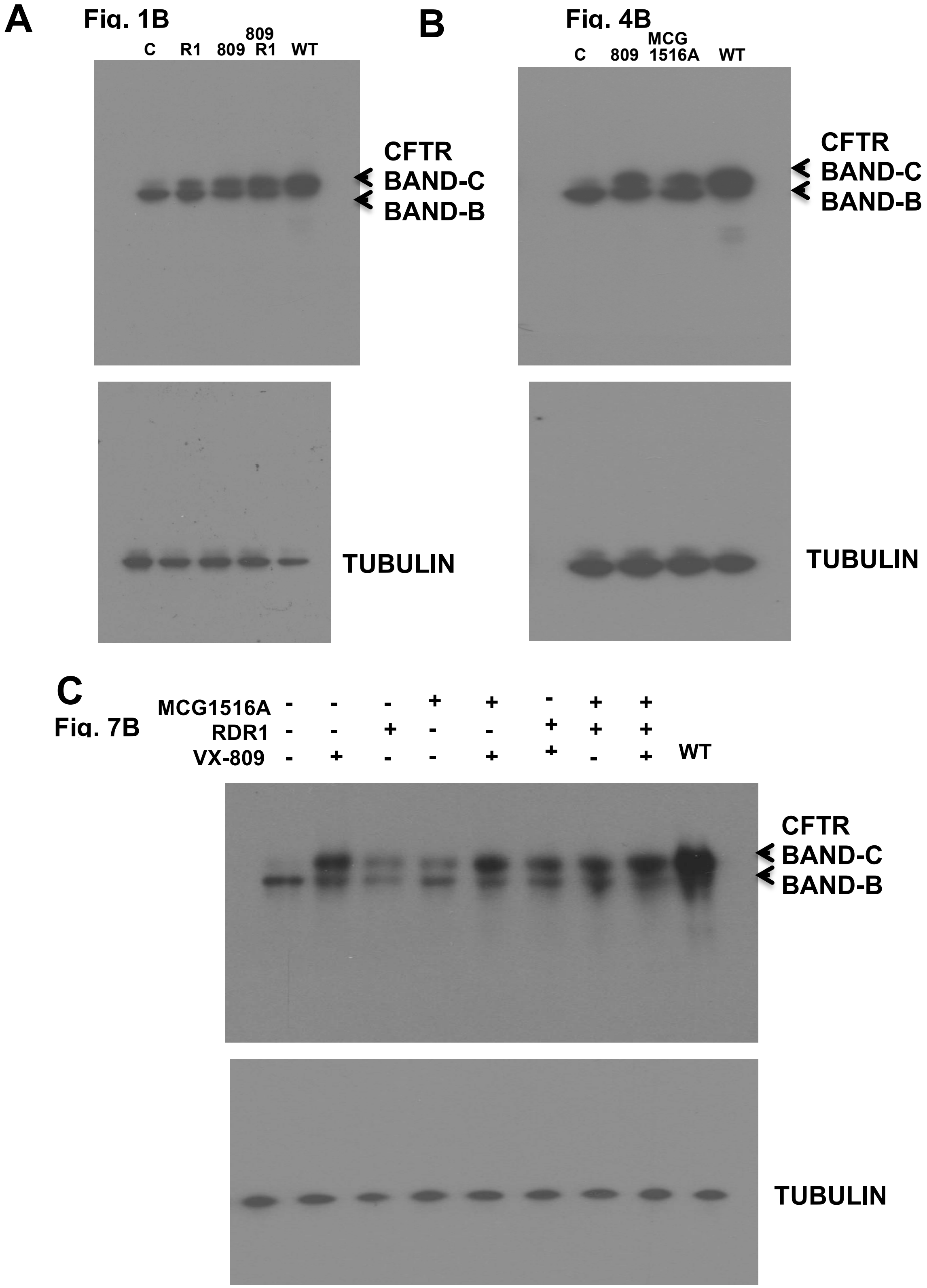

SUPPLEMENTARY FIGURE 1. Uncropped versions of western blot autoradiographs that appear in figures 1B (A), 4B (B), 7B (C) of the main text

SUPPLELEMENTARY FIGURE 2

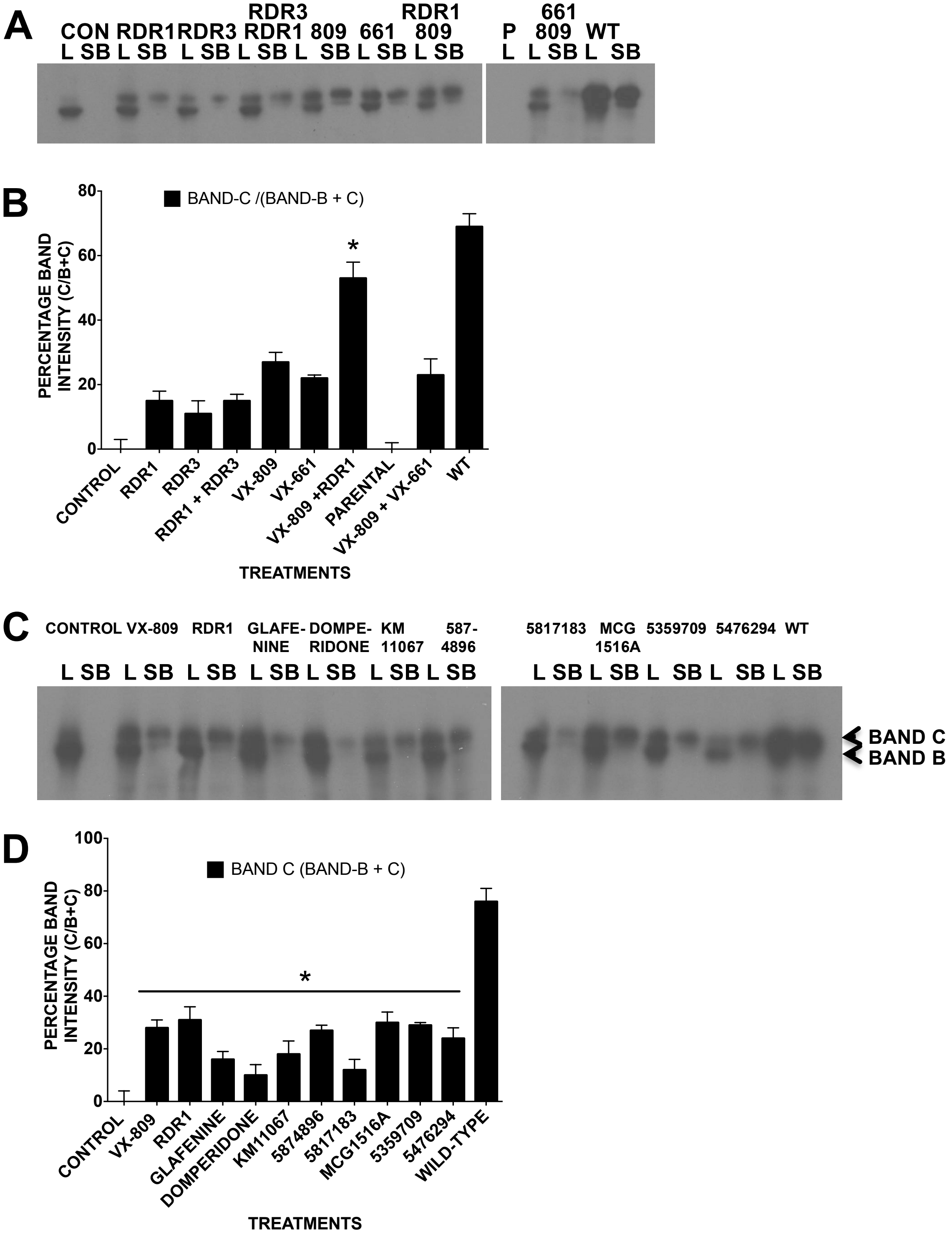

SUPPLEMENTARY FIGURE 2. Demonstration of F508del-CFTR being trafficked to the cell surface using surface biotinylation assays. A. Shows an immunoblot of lysates (L) and surface biotinylated fractions (SB) of BHK cells first treated for 24 hours with various correctors (VX-809 1μM, VX-661 1μM, RDR1 and RDR3 both 10μM). Also shown are wild-type CFTR expressing cells (WT) and parental BHK cells (P) not

expressing any CFTR. (B) A graph showing the relative intensity of the surface band C divided by the sum of the intensities of bands B and C in the lysate lane for the bands in part A. (C) Shows an immunoblot of lysates (L) and surface biotinylated fractions (SB) of BHK cells first treated for 24 hours with the correctors selected in figure 2 (see fig.2 and Sup. Table 1). (D) A graph showing the relative intensity of the surface band C divided by the sum of the intensities of bands B and C in the lysate lane for the bands in part C. Data in panels B and D are present as means ± SEM, $n = 4$, *, $p < 0.05$.

SUPPLEMENTARY FIGURE 3

A

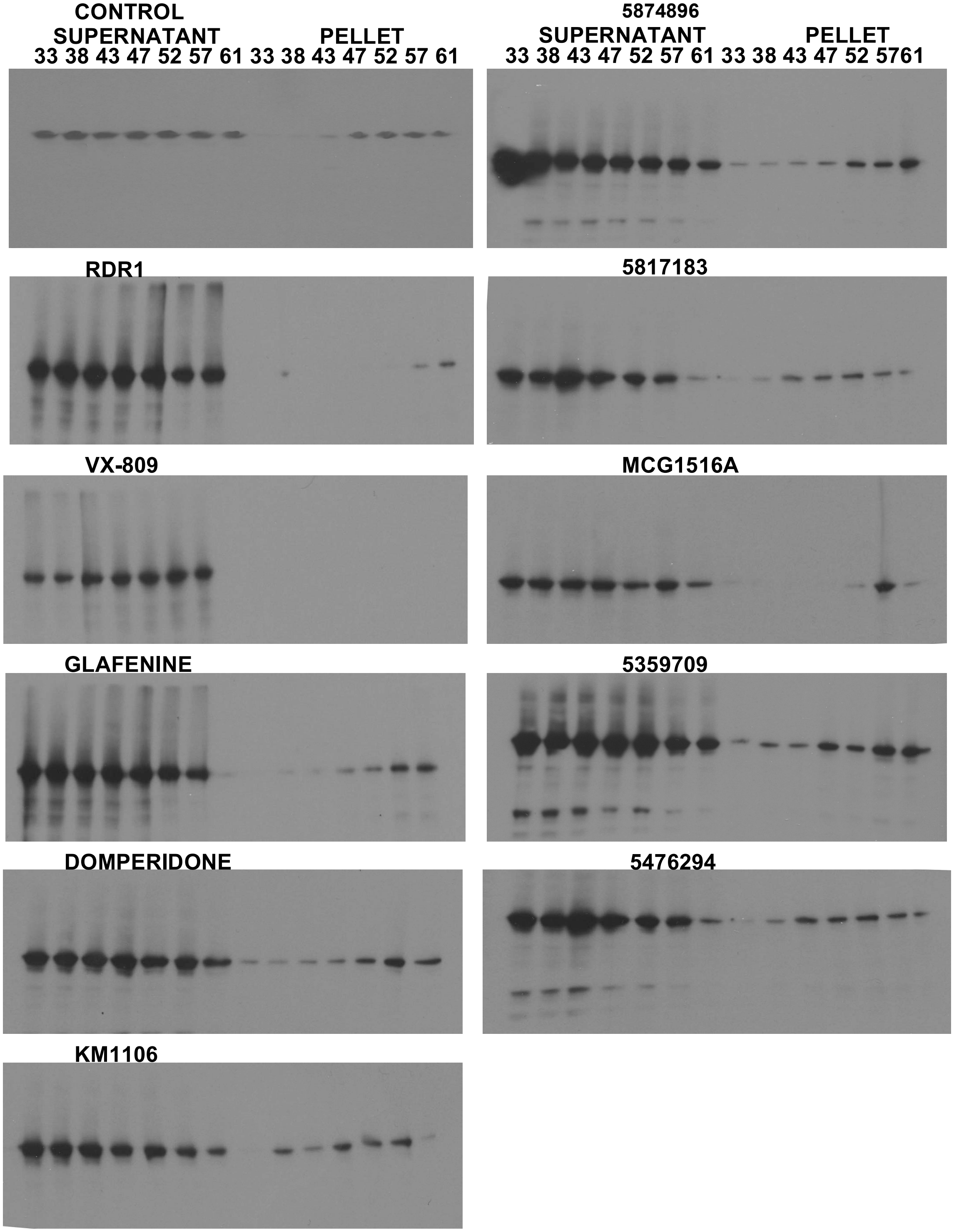

B

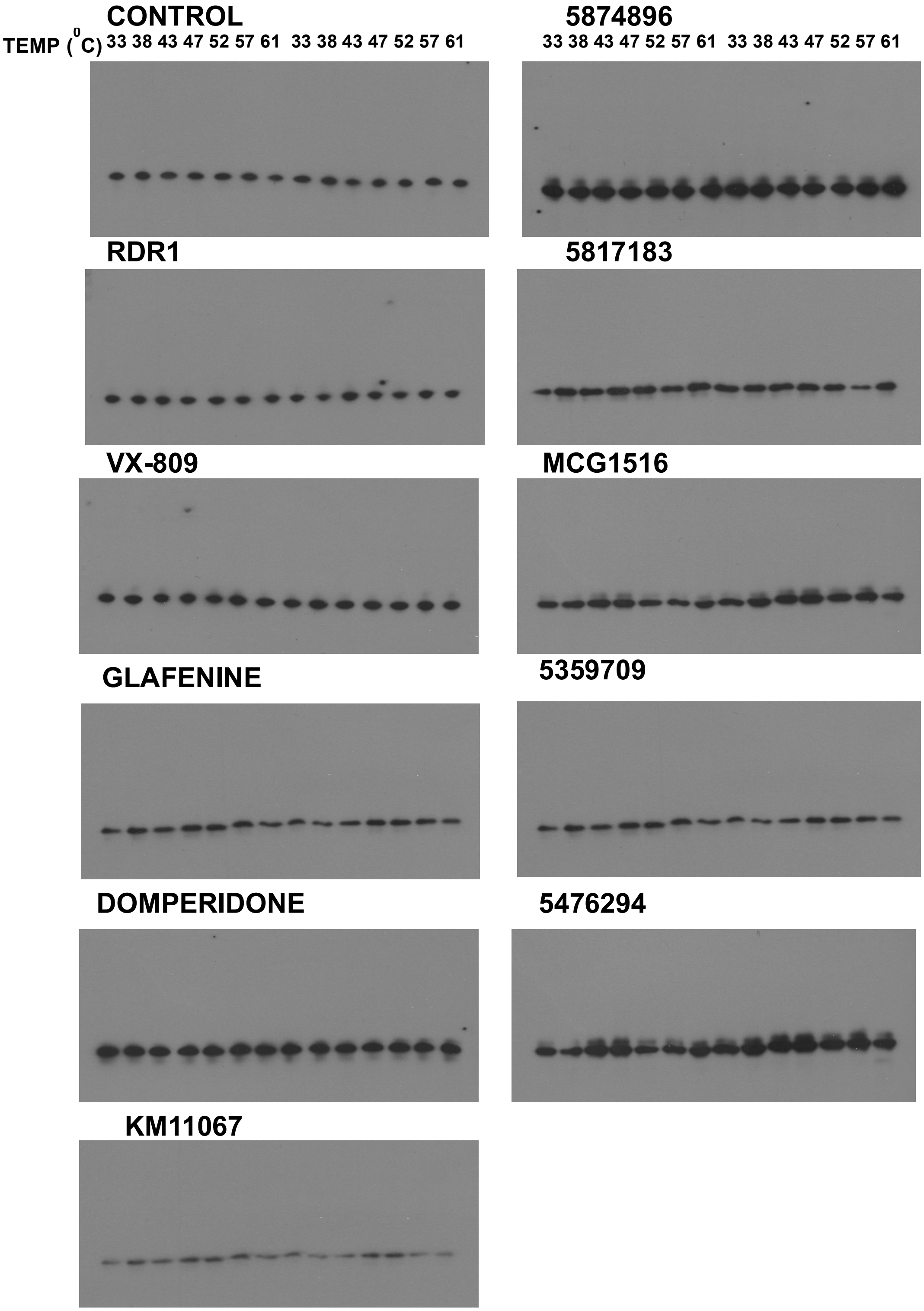

SUPPLEMENTARY FIGURE 3 (A) The figure shows the full uncropped versions of the western blot autoradiographs presented in figure 3 for each of the compounds tested in the CETSA assay (B) Shows the full autoradiograph of the tubulin protein loading control for the same blots.

SUPPLEMENTARY FIGURE 4

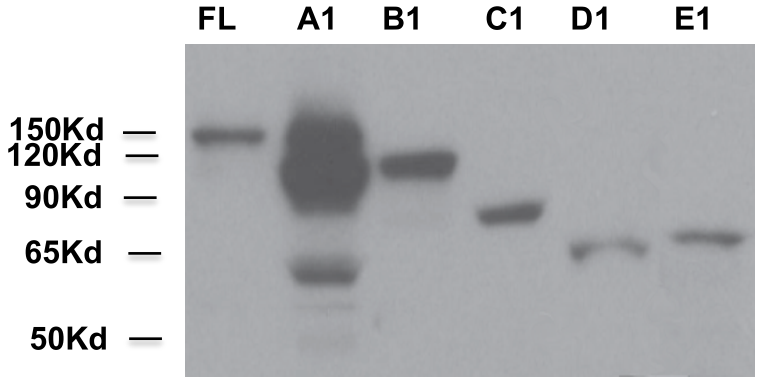

SUPPLEMENTARY FIGURE 4 Demonstration of the expression of F508del-CFTR deletion mutants in BHK cells by immunoblot. Each lane had 30μg of lysate

SUPPLEMENTARY FIGURE 5

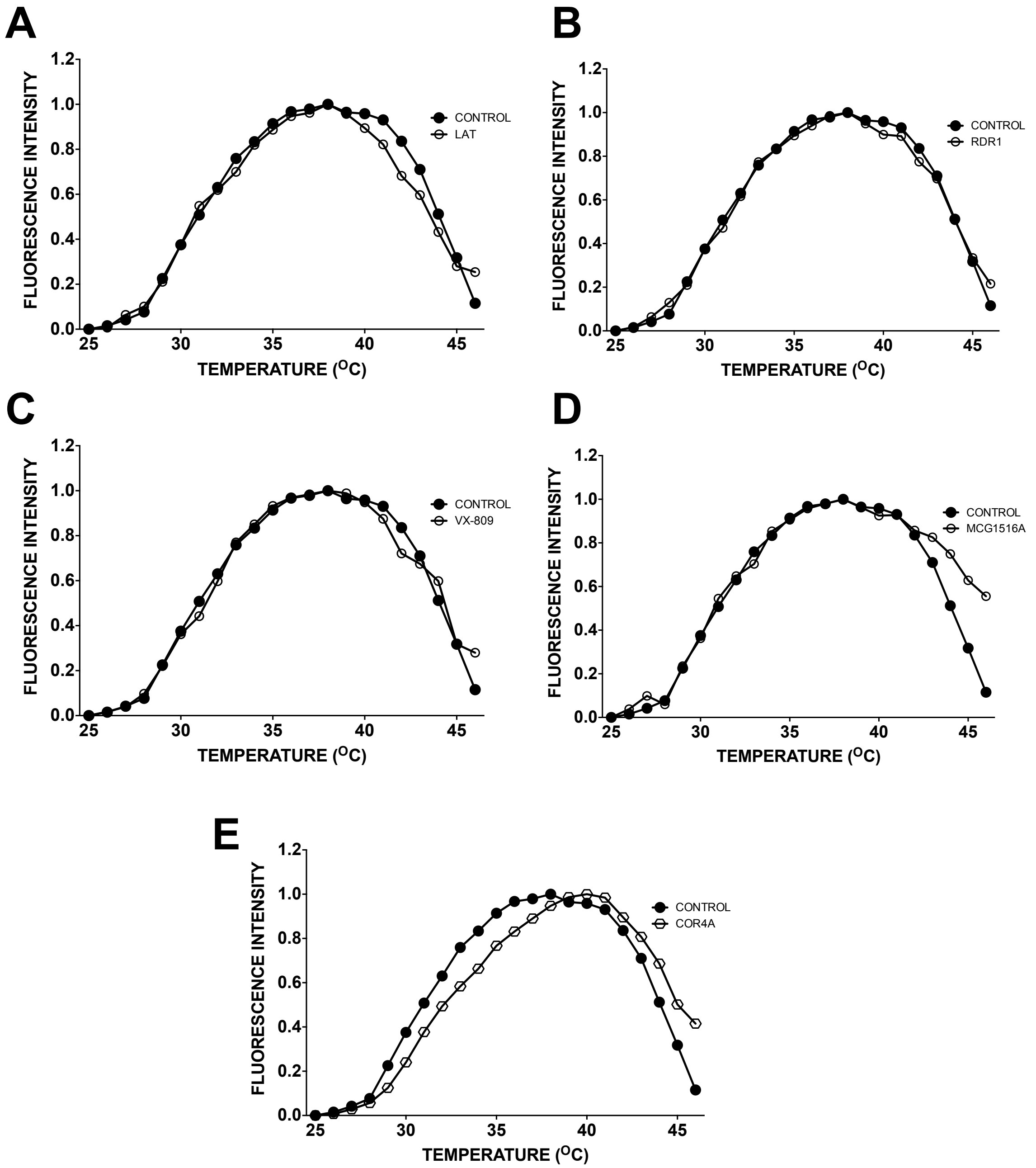

SUPPLEMENTARY FIGURE 5. Thermal stabilization of NBD2 in the presence of correctors. Representative fractional unfolding curves of CFTR-NBD-2 (expressed from a pET-SUMO-NBD2 SOL7 plasmid (1193–1445, Q1280E/H1402A/L1436D/Q1411D/Y1307N/S1255L/S1359A)) in the presence of (A) Latonduine (LAT) (10μM), (B) RDR1 (10μM), (C) VX-809 (1μM), (D) MCG1516A (1516a)(10μM), and (E) COR-4A (10μM).

SUPPLEMENTARY FIGURE 6

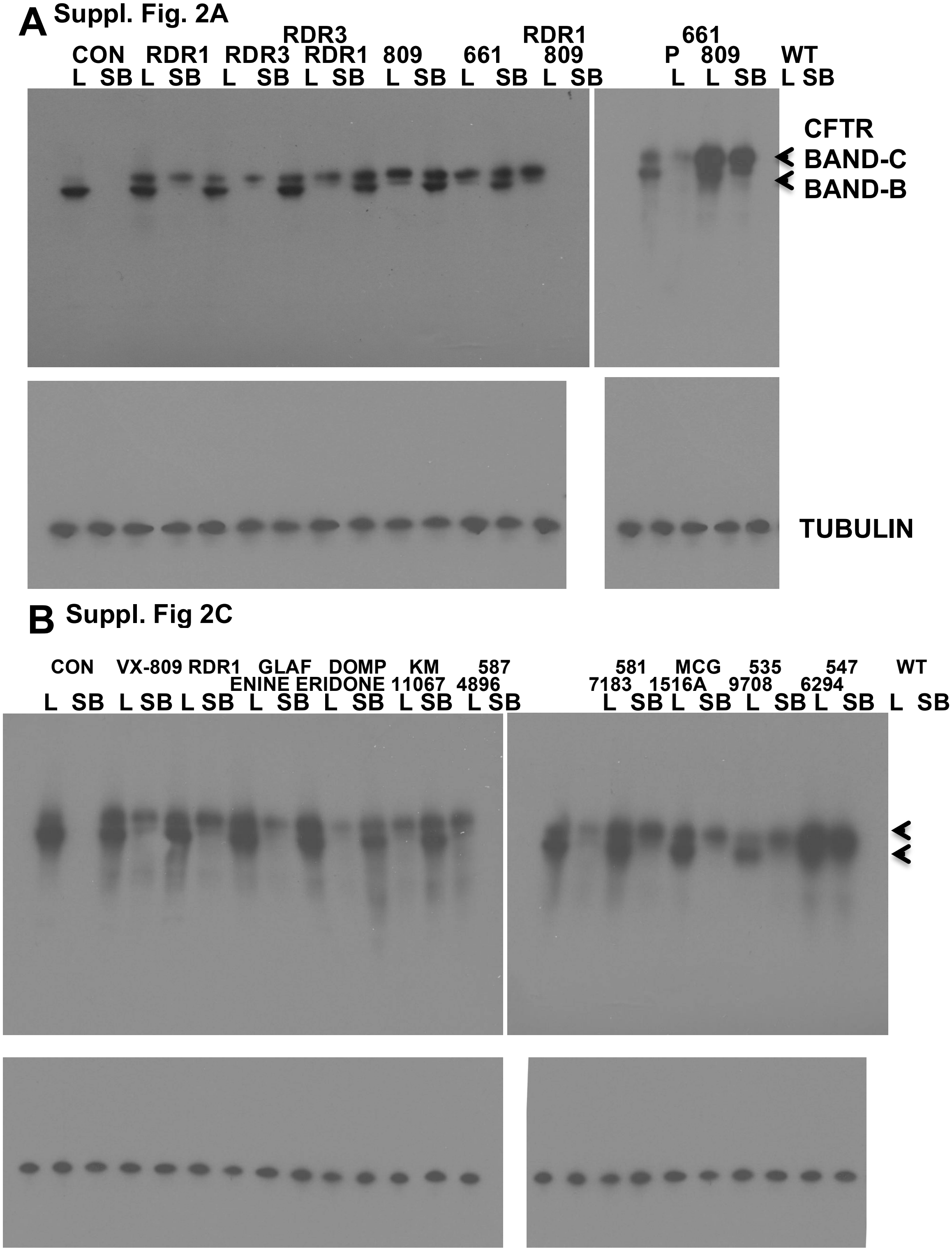

SUPPLEMENTARY FIGURE 6. Uncropped versions of western blot autoradiographs that appear in supplementary figure 2A (A) and 2C (B).

SUPPLEMENTARY FIGURE 7

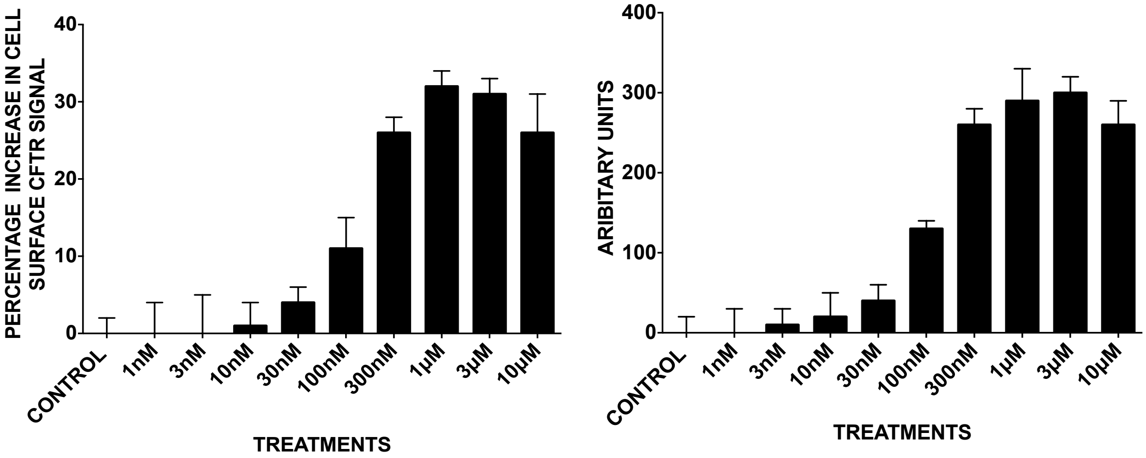

SUPPLEMENTARY FIGURE 8A

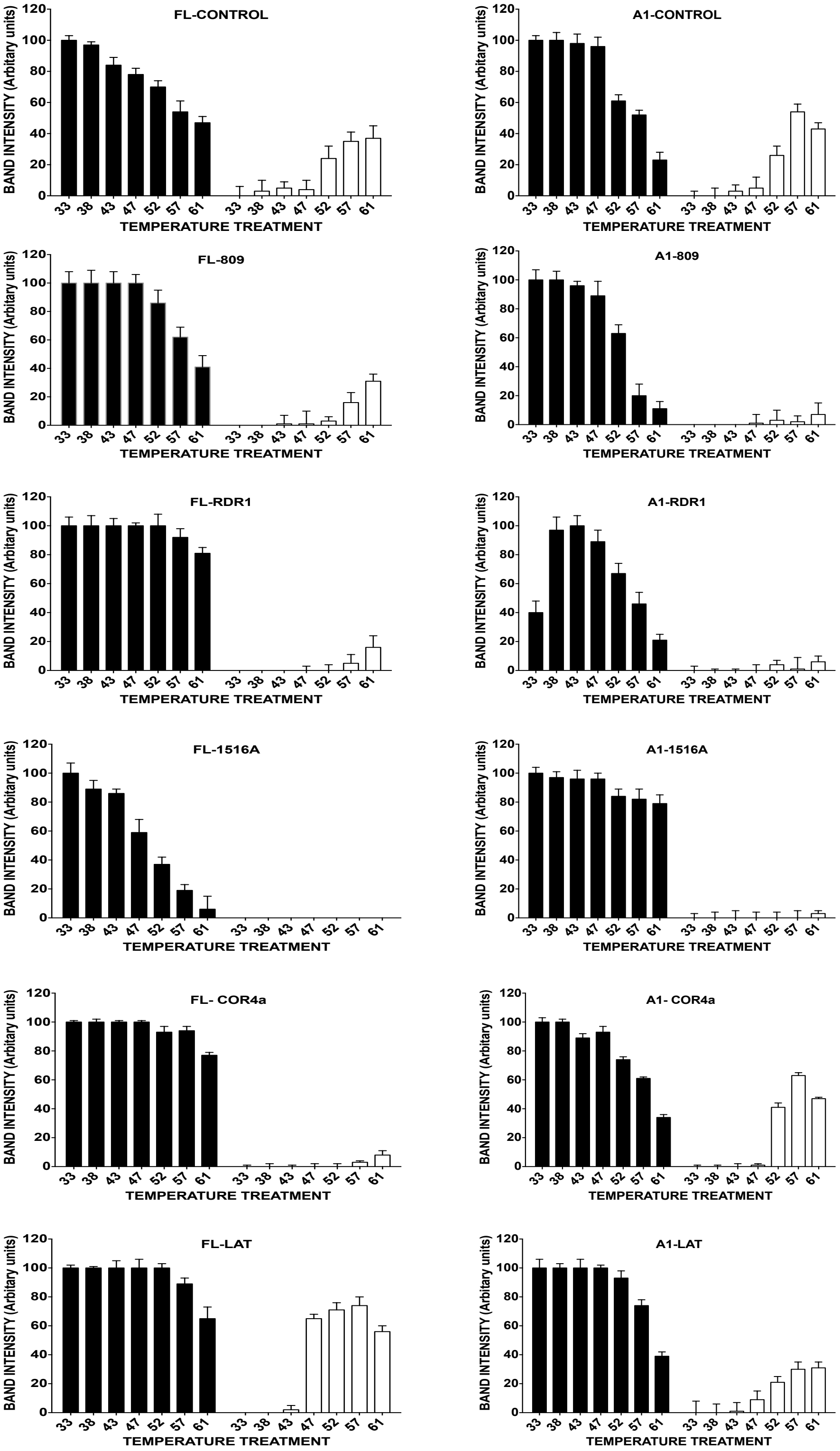

SUPPLEMENTARY FIGURE 8B

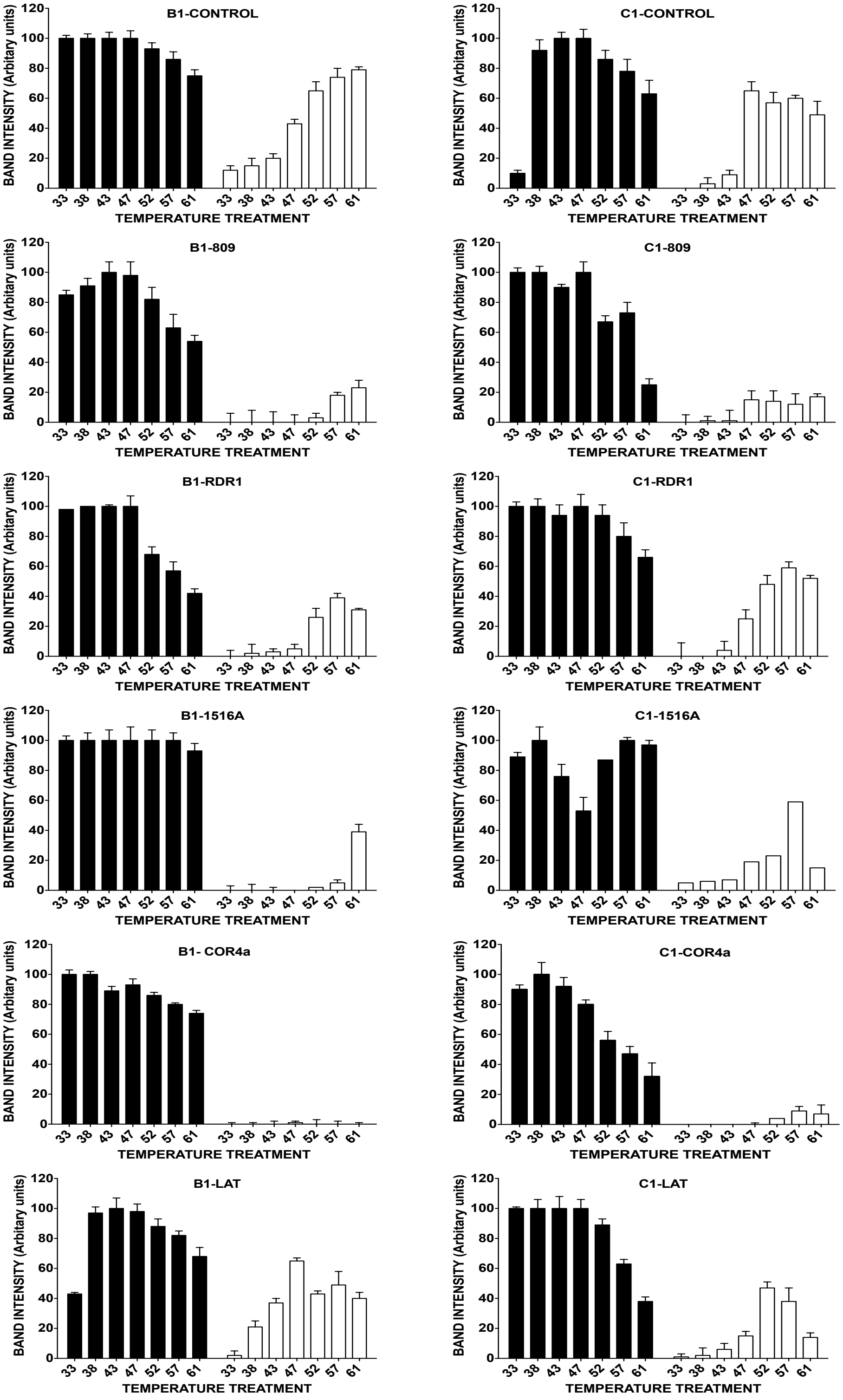

SUPPLEMENTARY FIGURE 8C

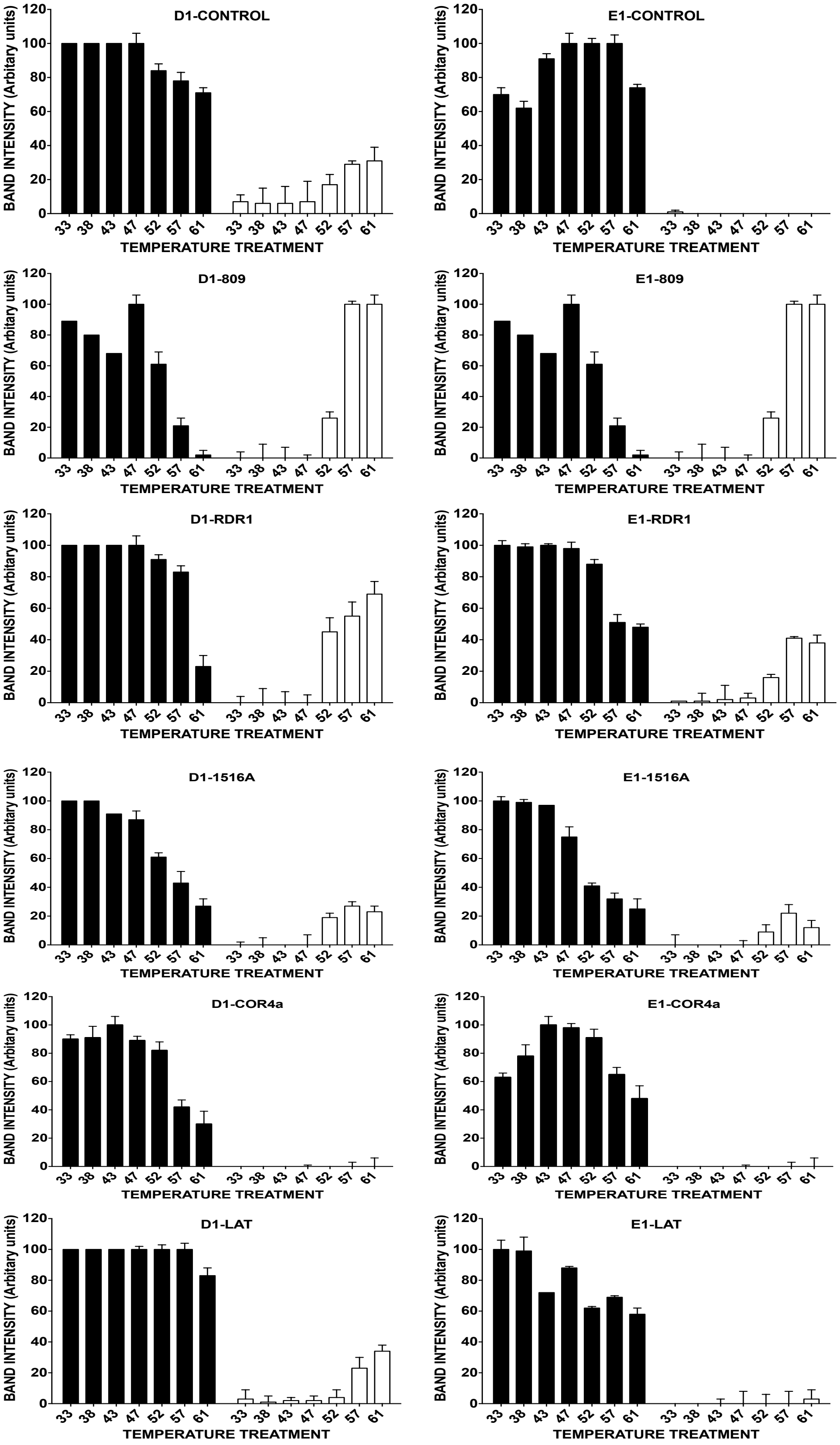

SUPPLEMENTARY FIGURE 7. Graphical representation of the CETSA results presented in Figure 5 and TABLE 1. Data in panels is present as means ± SEM, n = 4

SUPPLEMENTARY FIGURE 9

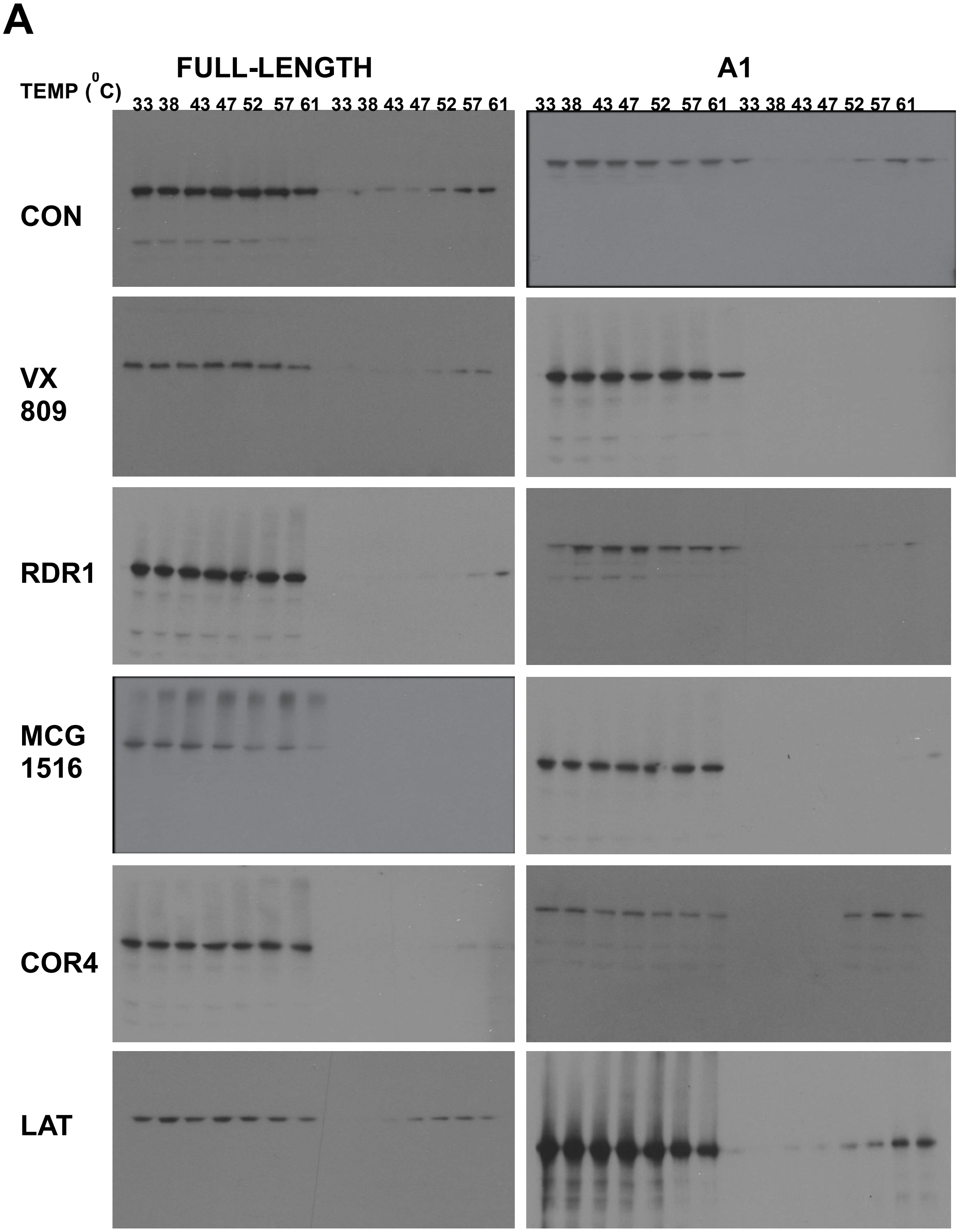

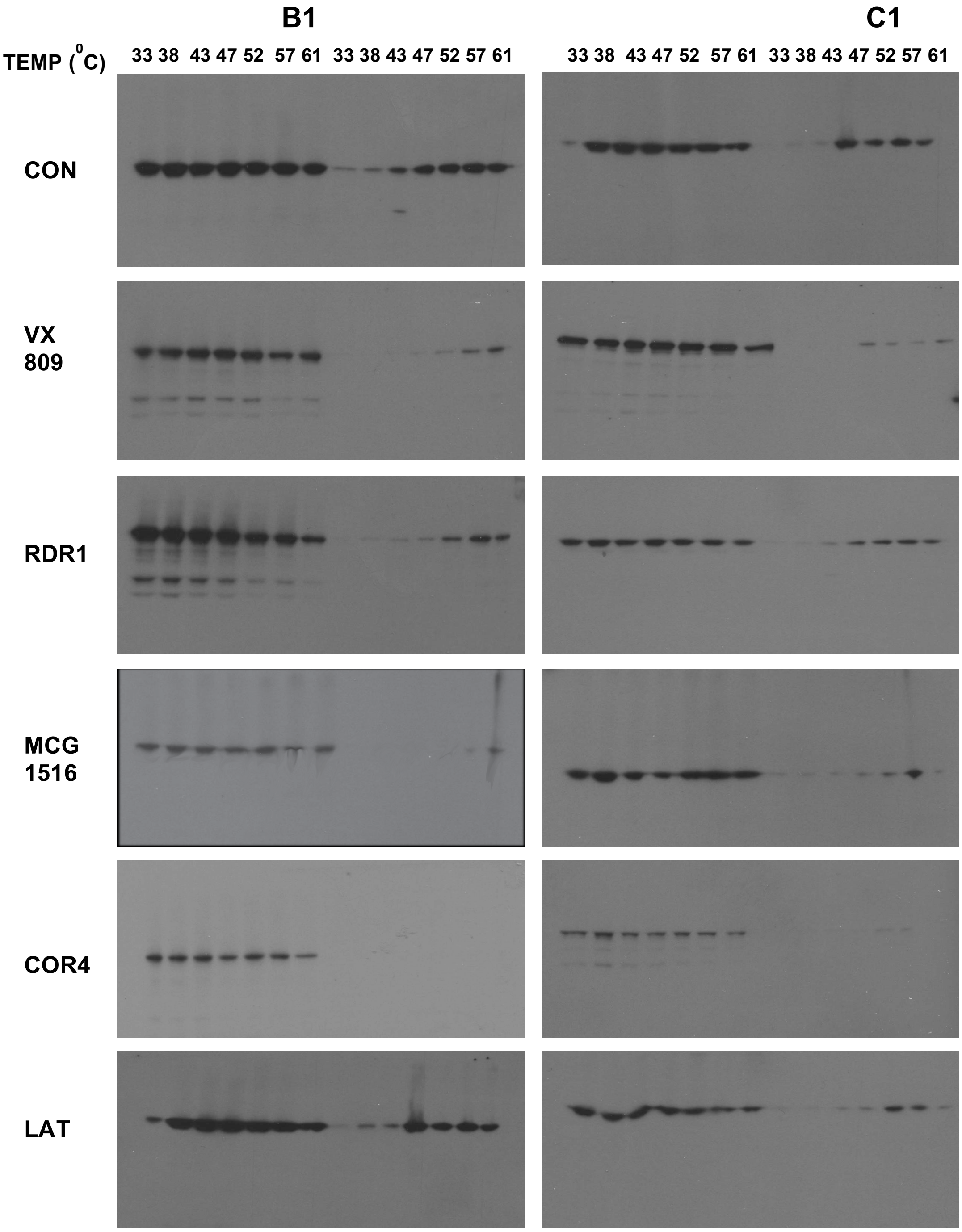
B1
C1
TEMP (0C)
33 38 43 47 52 57 61 33 38 43 47 52 57 61
33 38 43 47 52 57 61 33 38 43 47 52 57 61
CON
VX 809
RDR1
MCG 1516
COR4
LAT

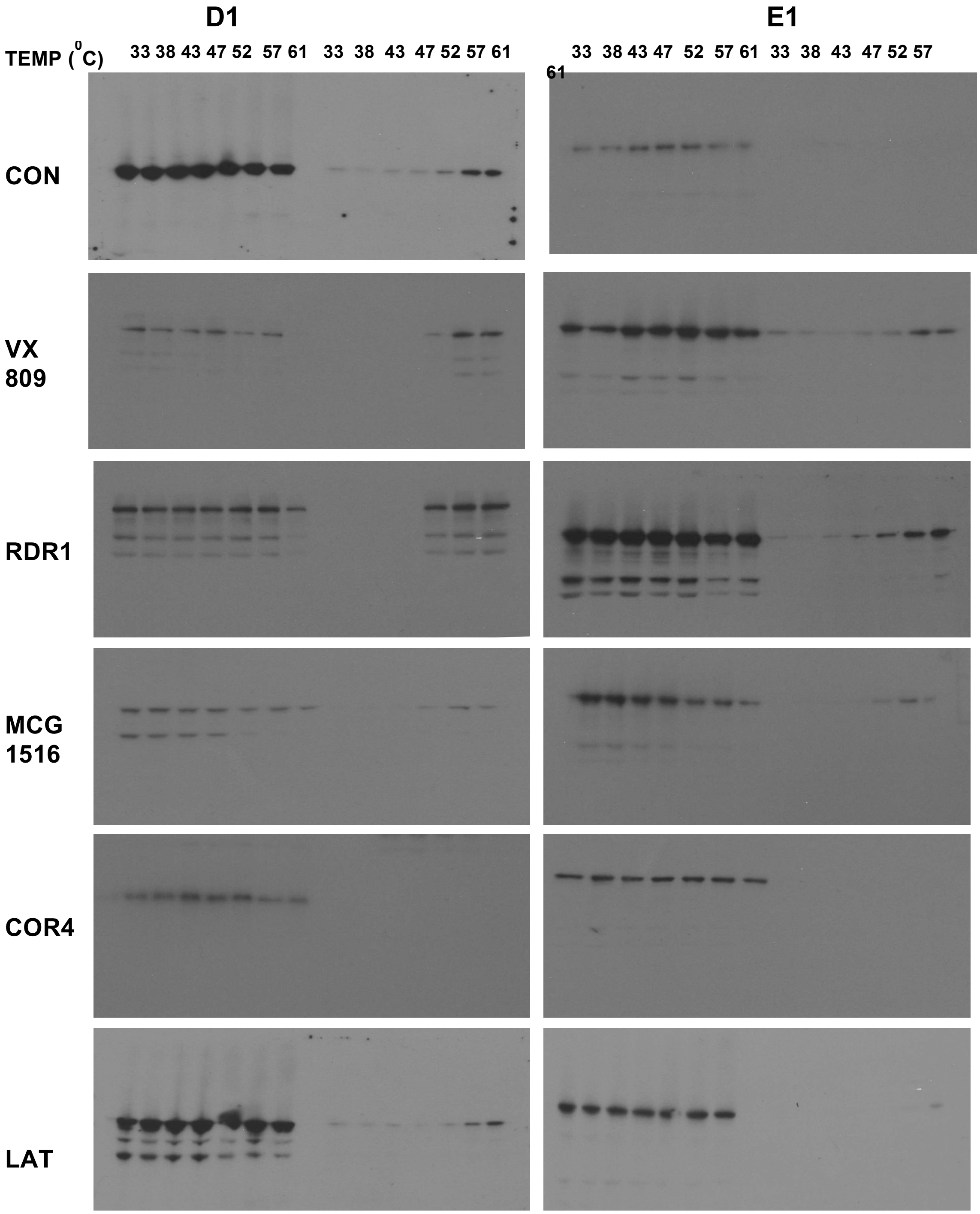
D1
E1
TEMP (0C)
33 38 43 47 52 57 61 33 38 43 47 52 57 61
33 38 43 47 52 57 61 33 38 43 47 52 57 61
CON
VX 809
RDR1
MCG 1516
COR4
LAT

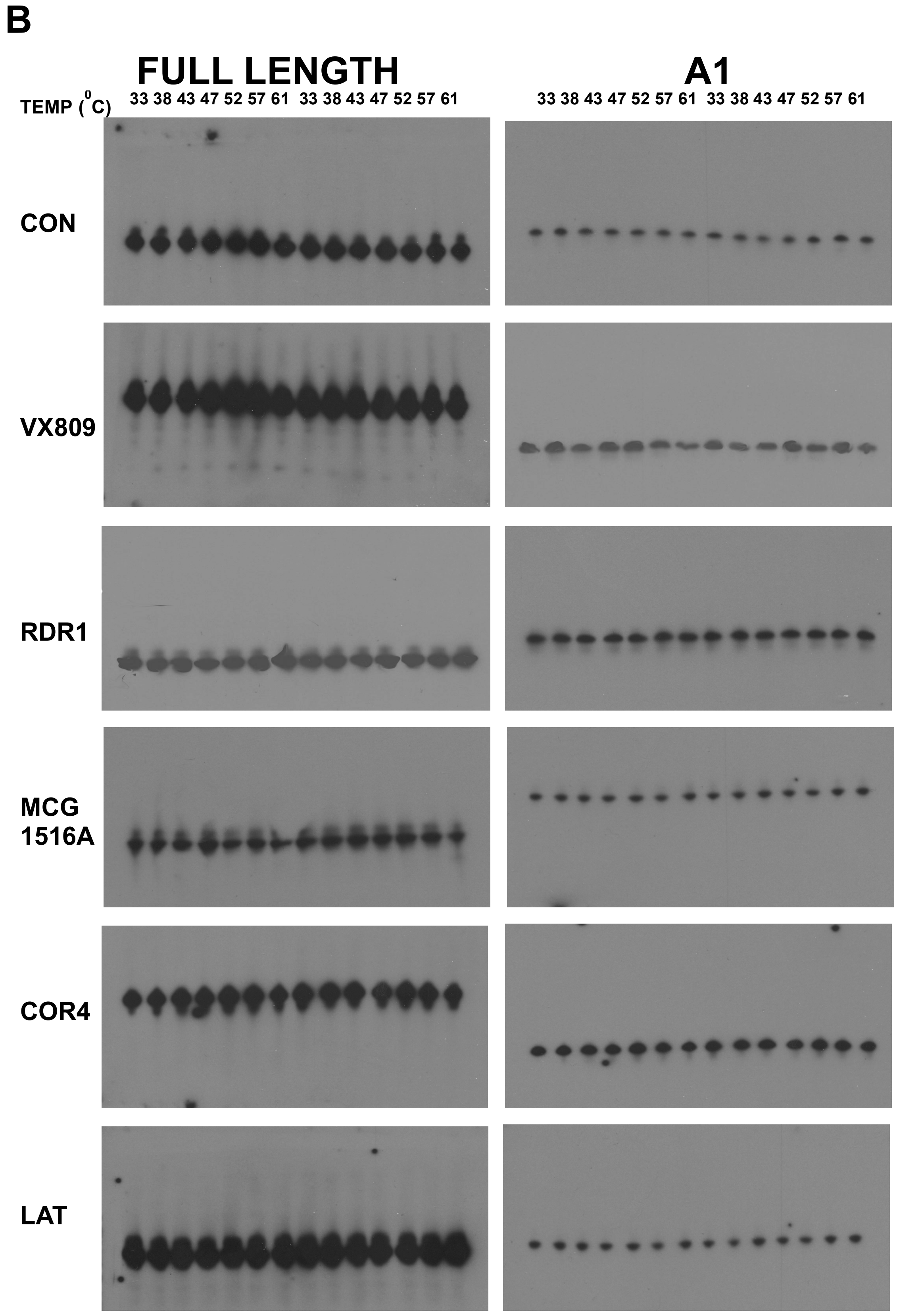
B
FULL LENGTH
A1
TEMP (⁰C)
33 38 43 47 52 57 61 33 38 43 47 52 57 61
33 38 43 47 52 57 61 33 38 43 47 52 57 61
CON
VX809
RDR1
MCG 1516A
COR4
LAT

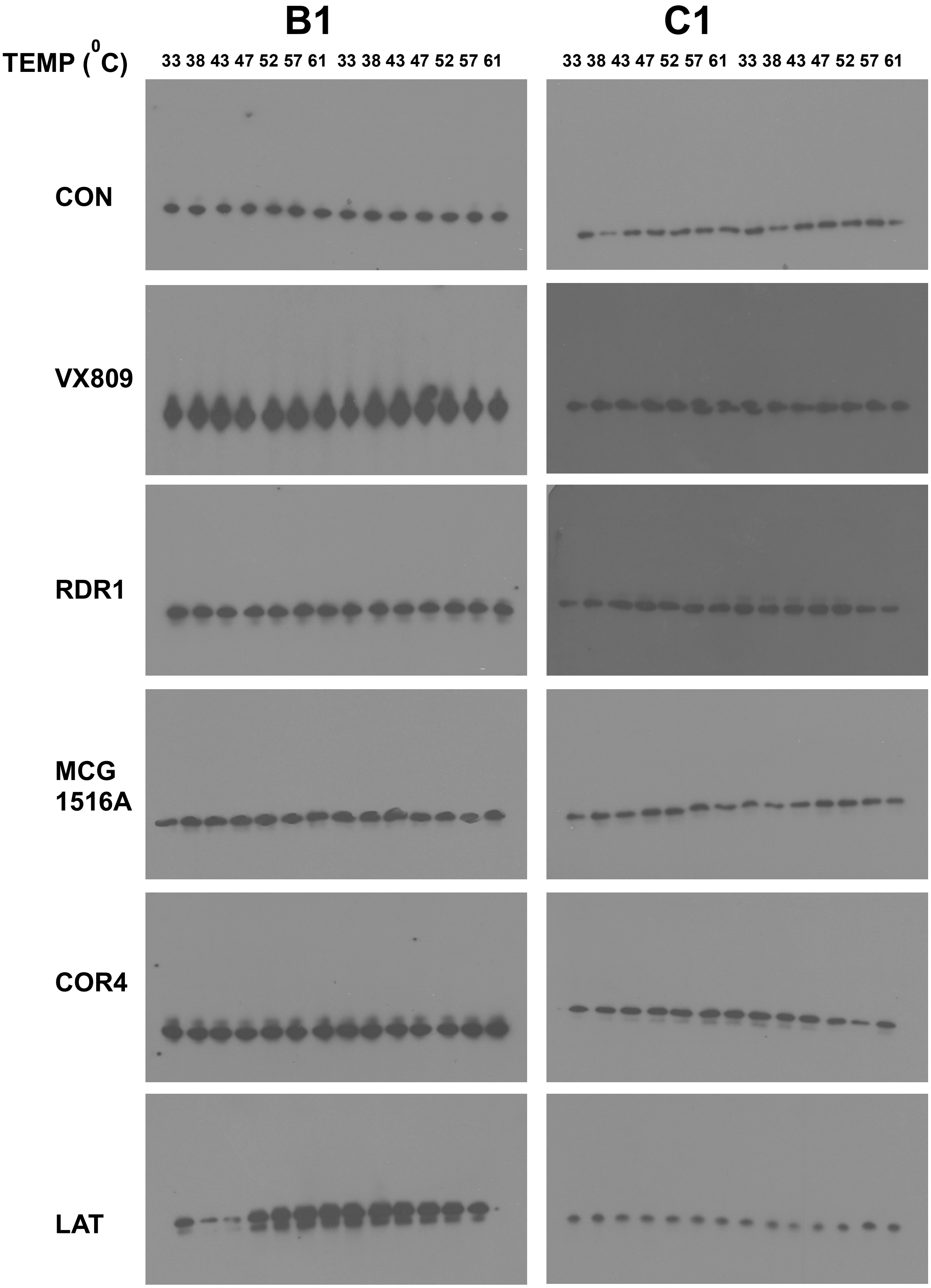
B1
C1
TEMP (^{0}C)
33 38 43 47 52 57 61 33 38 43 47 52 57 61
33 38 43 47 52 57 61 33 38 43 47 52 57 61
CON
VX809
RDR1
MCG 1516A
COR4
LAT

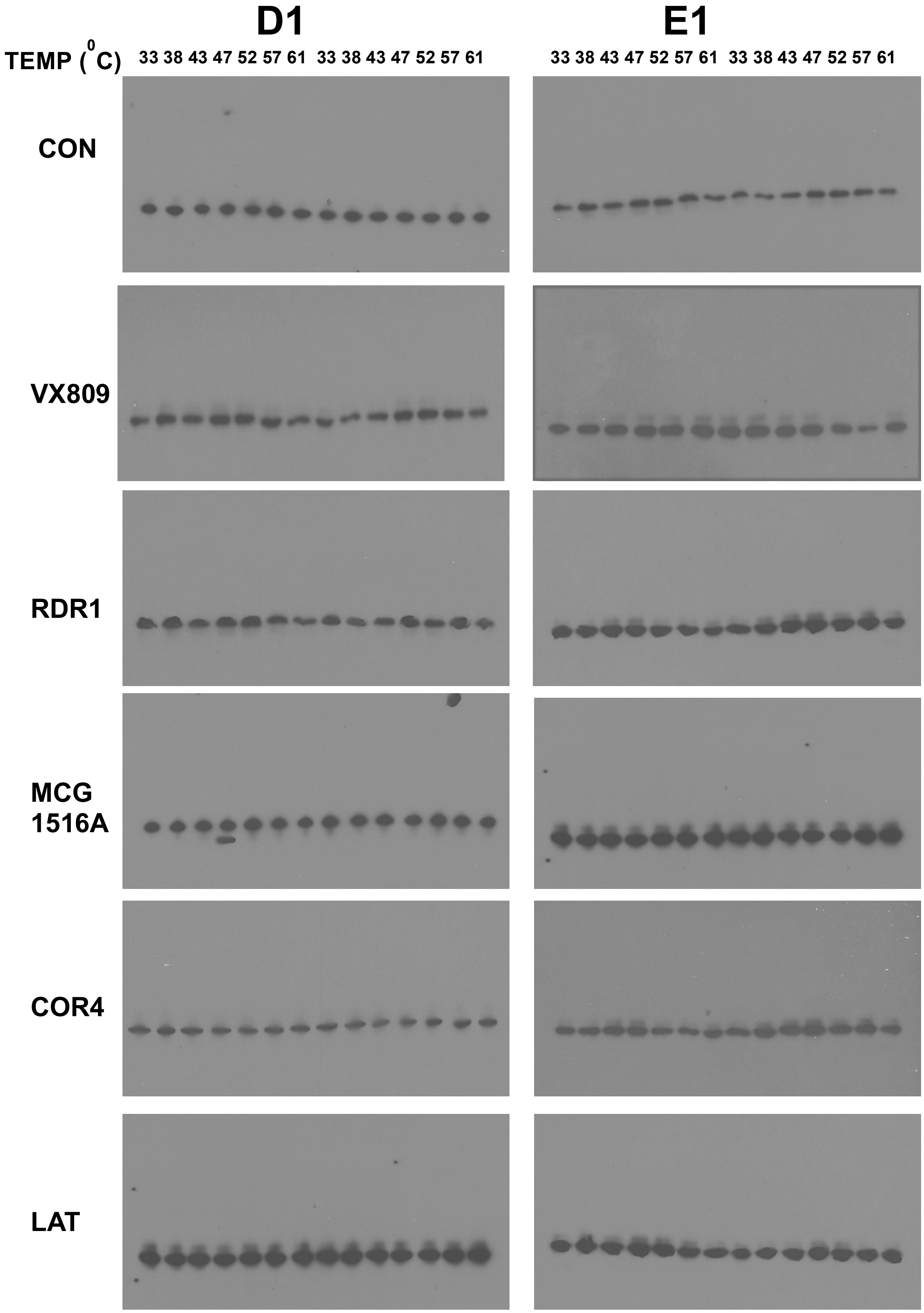

SUPPLEMENTARY FIGURE 9. (A) Uncropped versions of the western blot autoradiographs that appear in Figure 5. (B) Uncropped autoradiographs showing the same blots probed for loading control with an anti-tubulin antibody.

SUPPLEMENTARY TABLE 2

DRUGS	FULL LENGTH	1-1218 (A1)	378-1480 (B1)	679-1480 (C1)	835-1480 (D1)	1-689 (E1)
CONTROL	-	-	-	-	-	-
VX-809	+	+	+/-	-	-	+
RDR1	+	+	+	-	-	+
MCG1516A	+	+	+	-	-	+
COR4A	+	-	+	+	+	-
LATONDUINE	-	-	-	-	-	-

SUPPLEMENTARY TABLE 2. Results from Figure 5 presented in table form.
